# Enhanced meta ensemble stacking approach with XGBoost and optuna based detection of Parkinson's disease

**DOI:** 10.3389/fdgth.2026.1799144

**Published:** 2026-03-27

**Authors:** Annsley Mohan Joseph Raj, S. Lakshmi Kruthika, Abinaya S

**Affiliations:** School of Computer Science and Engineering, Vellore Institute of Technology, Chennai, India

**Keywords:** convolutional neural network (CNN), decision tree, meta-ensemble stacking, optuna, Parkinson's disease, random forest, support vector machine (SVM), XGBoost

## Abstract

Parkinson's disease (PD), a progressive neurological disorder affecting motor function, has been significantly rising in prevalence in recent years. Current diagnostic methods, relying on clinical observations, neurological exams, and periodical DaTscan imaging, may exhibit reduced sensitivity in the early stages. To develop a robust and multimodal machine learning model for early detection, an Ensemble Approach (ESDRCX) is proposed that integrates a meta-ensemble stacking technique that incorporates Decision Tree, Support Vector Machine (SVM) and Random Forest using quantitative data, along with a Convolutional Neural Network (CNN) for spiral image input. Additionally, the outputs are merged using XGBoost as the meta-learner optimized with Optuna-based Tree-structured Parzen Estimator (TPE). The ESDRCX attains a prominent 95.7% accuracy, 86% precision, 91% recall, 88.6% F1-score and 87% AUC with the HandPD dataset, denoting a significant progress in Parkinson's disease diagnostics. The proposed framework delivers an accurate, interpretable and computationally effective approach for early PD detection.

## 1 Introduction

Parkinson's disease (PD) is a neurodegenerative condition linked to the dysfunction of brain cells, contributing to a notable drop of 60%–80% in dopamine production, an essential organic compound important for regulating movement (1). This decline of dopamine results to a varied set of symptoms, integrating both motor and non-motor aspects. Motor symptoms comprise hallmark tremors, where involuntary shaking or trembling impacts the hands, fingers, or other parts of the body, especially at rest. Stiffness, an additional motor symptom, yields in muscle rigidity, causing limited movement and lowering the overall functional mobility. Problems in maintaining balance, termed as postural instability, can heighten the risk of injuries and falls. Apart from motor symptoms, PD also displays a spectrum of non-motor symptoms. Cognitive impairment, defined by difficulties in memory and attention, may occur, and in some cases, individuals may develop dementia (2). Autonomic dysfunction can lead to problems such as constipation, orthostatic hypotension and urinary problems. Additional factors that contribute to the varied nature of Parkinson's Disease include sleep and mood disorders like depression and anxiety, and a reduced sense of smell (hyposmia). Tremors, slowed movements and stiffness are examples that highlight the need for complex diagnostic tools propelling continued exploration into machine learning algorithms for PD prediction based on voice pattern analysis. Based on early detection, ESDRCX detects the significance of understanding and recognizing non-motor symptoms, safeguarding a holistic approach to improving the diagnosis and management of PD.

Inspired by prior works, the study utilized datasets adopting participant features, voice pitch measurements and sound analysis by employing the efficiency of the XGBoost algorithm (3, 4). Normalization and Synthetic Minority Over-sampling Technique (SMOTE) are advanced data preprocessing techniques that have proven fundamental for early PD detection (4). Conventional diagnostic techniques that predominantly rely on subjective medical assessments may lead to potential variability in diagnosis among medical specialists for the disease. However, there is a rising need to involve non-motor symptoms such as cognitive impairment and autonomic dysfunction into diagnostic protocols that reveal their importance in detecting disease progression. Further, this requires a transition towards adopting advanced machine learning (ML) techniques, such as deep learning (DL) and CNN to analyze a varied set of data sources including medical imaging modalities like PET and MRI scans, along with physiological signals namely electroencephalogram (EEG). By integrating these varied data streams, ML models open a potential direction to augment diagnostic accuracy and offer a more in-depth understanding of Parkinson's disease. Therefore, the advancement of innovative models that seamlessly combine these approaches becomes vital to overcome the constraints inherent in current diagnostic methodologies.

Various limitations exist despite extensive research on machine learning based PD detection. Existing studies focus on single-modality data like voice, wearable signals that either require expensive infrastructure or limits feature diversity. Further, deep learning models rely on large datasets and highly computational resources which reduce stability. Numerous ensemble methods lack systematic hyperparameter optimization, increasing risk of overfitting. To address these limitations, ESDRCX introduces an innovative diagnostic system that utilizes a fusion-based machine learning solution. This method uses Decision Tree, SVM, Random Forest and CNN in their conventional forms, that includes examining quantified data as well as hand-drawn spiral patterns from images to optimize the sensitivity and accuracy of PD detection, notably in its early stages. The models mentioned were chosen as they demonstrate marked benefits that help with the improvement of the overall model. The core methodology of the proposed system implements a meta-ensemble stacking technique, which is an advanced machine-learning model hybrid approach. The technique is enhanced further using XGBoost with hyperparameter optimization, facilitated by the Optuna-based TPE framework because it exhibited an increase in the overall model accuracy. The optimization process guarantees that the model tries to attain the highest possible accuracy related to Parkinson's disease while ensuring a lesser chance for overfitting. The proposed model achieves an accuracy of 95.7%, constituting a substantial advancement in Parkinson's disease diagnostics. The previous ensemble models lack dynamic optimization or operating on a single input stream. The novelty of ESDRCX lies in the incorporation and fusion of dual input streams (quantified handwriting features and spiral images) through a meta-stacked ensemble framework, and in the systematic Optuna–TPE-based hyperparameter optimization of the meta-learner to enhance robustness and generalization. This integrated design leads to better class balance and higher diagnostic accuracy across both internal and external datasets, differentiating it from conventional single-layer or image-only models. The approach not only supports early detection but also establishes a benchmark for future innovations in the field of neurodegenerative disorder diagnostics.

Beyond the domain of Parkinson's disease analysis, broader methodological advancements in simulation, engineering education, and lightweight computer-vision architectures have formulated contemporary computational modelling trends. Simulation-based learning in structural and mechanical systems (5), curriculum-based frameworks focused on the domain of engineering education (6), and efficient object-detection models for real-time setting (7) are few of the distinctive examples involved. The growing attention on computational efficiency, scalable learning approaches and structured model architecture underpins the modern applied study.

The key findings of the proposed Ensemble Approach (ESDRCX) include the following:

1. Dual inputs are employed with hand-drawn spiral images and quantified data which are observations retrieved from the same images, that enhances diagnostic accuracy and provides deeper understanding of Parkinson's Disease.
2. The method of Meta Ensemble Stacking with XGBoost in the ESDRCX framework is incorporated and yields thorough analysis via XGBoost as a meta-learner, thus improving analytical accuracy in view of diverse perspectives.
3. Optuna-based Tree-structured Parzen Estimator (TPE) effectively optimizes the hyperparameters of the proposed ESDRCX methodology, that further improves the robustness of the model.
4. Experimental evaluation demonstrates the superiority of TPE with Optuna incorporated with Meta Ensemble Stacking with XGBoost in the ESDRCX framework, verified by evaluation techniques and key metrics, highlighting its potential for clinical applications and constituting an advancement in Parkinson's disease diagnosis.

**Figure 1:**
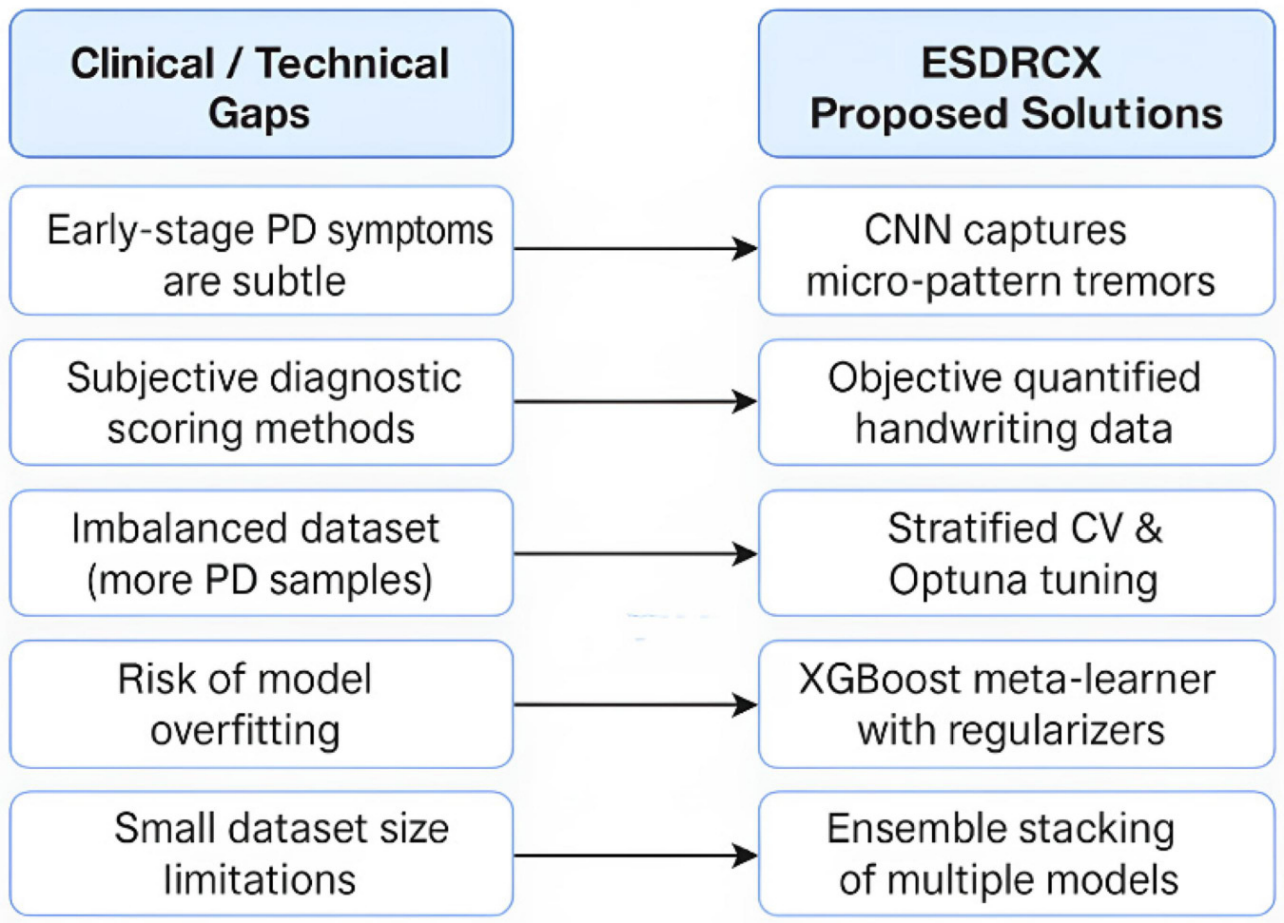
Summary of design motivations.

To contextualize the proposed framework, Figure 1 depicts how key clinical and technical limitations in PD diagnosis are examined through specific design components in ESDRCX.

The organization of the paper after introduction is as follows. Section 2 discusses the literature survey on machine learning, deep learning, hybrid and ensemble approaches for Parkinson's disease detection. Section 3 shows the proposed methodology including preprocessing, base learners, meta-ensemble stacking, XGBoost with Optuna based TPE optimization. Section 4 presents the experimental evaluation including the dataset description, baselines, evaluation metric, experimental setup, results and discussions, generalizability and fairness and demographic bias evaluation. Section 5 concludes the paper with conclusion and future works.

## 2 Literature survey

The advancement of the Parkinson's Disease raises challenges to the central nervous system that affects various motor functions of the body (3). To improve PD detection models, research focusing on feature selection, ensemble learning methods and genetic algorithms emphasizes the multifaceted approaches applied (8). Multiple base classifiers sensitive to distinct subsets of features and samples are combined by recent research results of the model, offering an improved approach for PD detection (9). This aspires to advance the field by providing deeper understanding of early discovery. To address the PD challenges that play a vital role in telemedicine, the study implements machine learning models such as SVM, Random Forest, KNN and Logistic Regression to remotely detect PD using MDVP audio data (10). The attention on the significance of the Random Forest model within telemedicine underscores the addition of machine learning. The assessment of voice disorders as a diagnostic marker deepens as the research enhances techniques for early PD diagnosis. This is proposed using novel methods like SMOTE, RFE and dimensionality reduction through t-SNE and PCA (1). PCA is applied to address multicollinearity issues that has proven to be effective in approximating UPDRS scores for both Motor and Total-UPDRS. The scope of Parkinson's Disease (PD) research expands upon deriving facets beyond traditional motor symptoms. Machine learning plays a central role in the development of PDD-ET (11), an advanced model for early-stage PD detection. By using various patient data, including mobility, medication habits, mobility and medical history, PDD-ET marks a substantial progress towards more accurate and stable tools for early PD diagnosis. In the realm of personalized medicine (12), the merging of machine learning and multimodal data takes a transformative change. GenoML, an automated ML package, is implemented to enhance various predictions of PD risk, justified across external cohorts. The proposed methodologies contribute to the development of PD diagnostic tools focusing on practicality and efficiency.

The research further develops on the deep learning foundations, aiming to support the emerging landscape of PD detection methodologies. Further, this adds insights from multiple approaches involving ensemble techniques and feature selection integration with deep neural networks to formulate an effective predictive model for early PD detection through voice analysis (9, 13). Additionally, the study extends its impact by introducing CNN for detecting PD patients from healthy controls derived from drawing tasks (12) presenting potential real-time diagnostic applications within medical environments. Advancing into the neurodegenerative features of PD, additional studies delve into the DL ensemble method for predicting PD using DaTscan images (14). VGG16 and Xception are embedding models that outperform individual models in performance. The user-friendly GUI-based software tool presented holds considerable potential for real-time PD detection. The research applies a DNN model using a reduced input feature space by studying the prediction of PD development (15).

Ensemble techniques implemented via Nearest Neighbor Boosting (NNB) have demonstrated higher performance by outdoing the baseline models in key metrics (13). A stacking methodology is introduced in (16) for enhanced PD classification but is restricted to structured feature inputs without multimodal fusion. An innovative proof-of-concept model utilized in (17) shows cardiac electrical activity analysis to detect subjects at high risk of PD development by exploring autonomic nervous system involvement. The unique approach adds complexity to the understanding of PD risk factors extending beyond classical heart rate variability metrics. Transitioning to early-stage diagnostics (18), a non-invasive procedure helps to differentiate PD from vital tremors by examining the autonomic dysfunction via skin vasomotor response to cold stimuli. A hybrid framework is proposed in (19), which shows the value of incorporating spatial and sequential representations in a combined architecture. The gain of fusing multiple imaging modalities with explainable machine learning modules is highlighted (20). A non-invasive Parkinson's disease progression model using voice signal analysis was proposed by (21). It shows acoustic features like pitch and jitter that act as reliable biomarkers for early PD detection. With the introduction of an artificial intelligence (AI) system, the field of PD assessment has experienced technological advancement (22). The system utilizes standardized finger-tapping tasks designed for remote evaluation to systematically examine motor performance in patients with PD. Traditional stacking approaches combine homogeneous models or depend on single-modality inputs and fixed meta-classifiers. The proposed approach integrates dual-modality fusion of quantified handwriting features and CNN-based spiral images. Further, the enhanced meta-ensemble framework incorporates heterogeneous models across dual modalities and includes an Optuna-TPE optimized XGBoost meta-layer. The fusion without Optuna recorded accuracy of 94.7% while the TPE-based optimization increases the accuracy to 95.7%. These quantified improvements facilitate improved performance and systematic hyperparameter tuning.

An explainable convolutional neural network is introduced by (23) for Parkinson's disease detection. This incorporates visualization methods to showcase clinically related regions affecting model's results. In addition, this highlights interpretability in medical imaging-based diagnosis forwarding the need for transparency in AI driven healthcare. Existing limitations in data diversity, model generalization and clinical translation are identified in (24) for early Parkinson's disease detection. This highlights the future research for scalable PD detection systems.

Although innovative advancements are present, various limitations are present in existing PD detection. These include dependence on single-modality data like voice, imaging, inadequate systematic hyperparameter optimization, limited importance on explainability of ensemble frameworks and minimal search of decision-level merging strategies. The novel use of technology presents and broadens avenues for prevalent and accessible PD assessment. A concise comparison is given below in Table 1.

**Table 1:** Literature review.

Reference Number	Methodology	Inference
(3)	XGBoost	Limited use of individual machine learning algorithms
(4)	XGBoost	Reliance on a single machine learning algorithm (XGBoost)
(8)	Ensemble Models	Incorporates genetic selection for feature selection (potentially less advanced)
(13)	k-Nearest Neighbor (k-NN) and Gradient Boosting (GB)	The highest accuracy received was approx. 74% after comparison with 11 other models.
(9)	Feature selection with deep neural networks (DNN)	Possibility of overfitting with extremely high accuracies
(10)	Logistic Regression, SVM, Random Forest Regressor, and KNN	Reliance solely on audio data for PD detection
(1)	Support-vector machine (SVM), K-nearest neighbors (KNN), decision tree (DT), random forest (RF), and multilayer perceptron (MLP)	Limited evaluation of the complete presentation.
(12)	CNN	Proof-of-concept nature, potentially limiting generalizability
(14)	Deep Learning (VGG16, ResNet50, Inception-V3, and Xception)	Reliance solely on DaTscan images for PD diagnosis, focusing primarily on motor symptoms
(15)	DNN	Focus on predicting scores, primarily assessing motor symptoms.
(17	Probabilistic Symbolic Pattern Recognition, Logistic Regression	Higher chance of generalizability
(18)	K-means clustering	Limited use of ML algorithm with small sample size and higher chance of cultural penmanship
(22)	LightGBM Regressor	Limited to assessing motor symptoms remotely using a webcam and finger-tapping task, which may not capture the full spectrum of PD symptoms
(11)	SVR, CNN, Stacked LSTM, Decision Tree, RF, and Deep Neural Network	Potential overfitting due to the use of a highly specific dataset tailored for early-stage PD detection, which may not fully represent the variability in real-world PD cases
(12)	12 ML Algorithms	Limited validation of the model's performance in an external cohort, potentially affecting its generalizability

## 3 Proposed methodology—meta ensemble stacking integrated XGBoost using optuna (ESDRCX) framework

**Figure 2:**
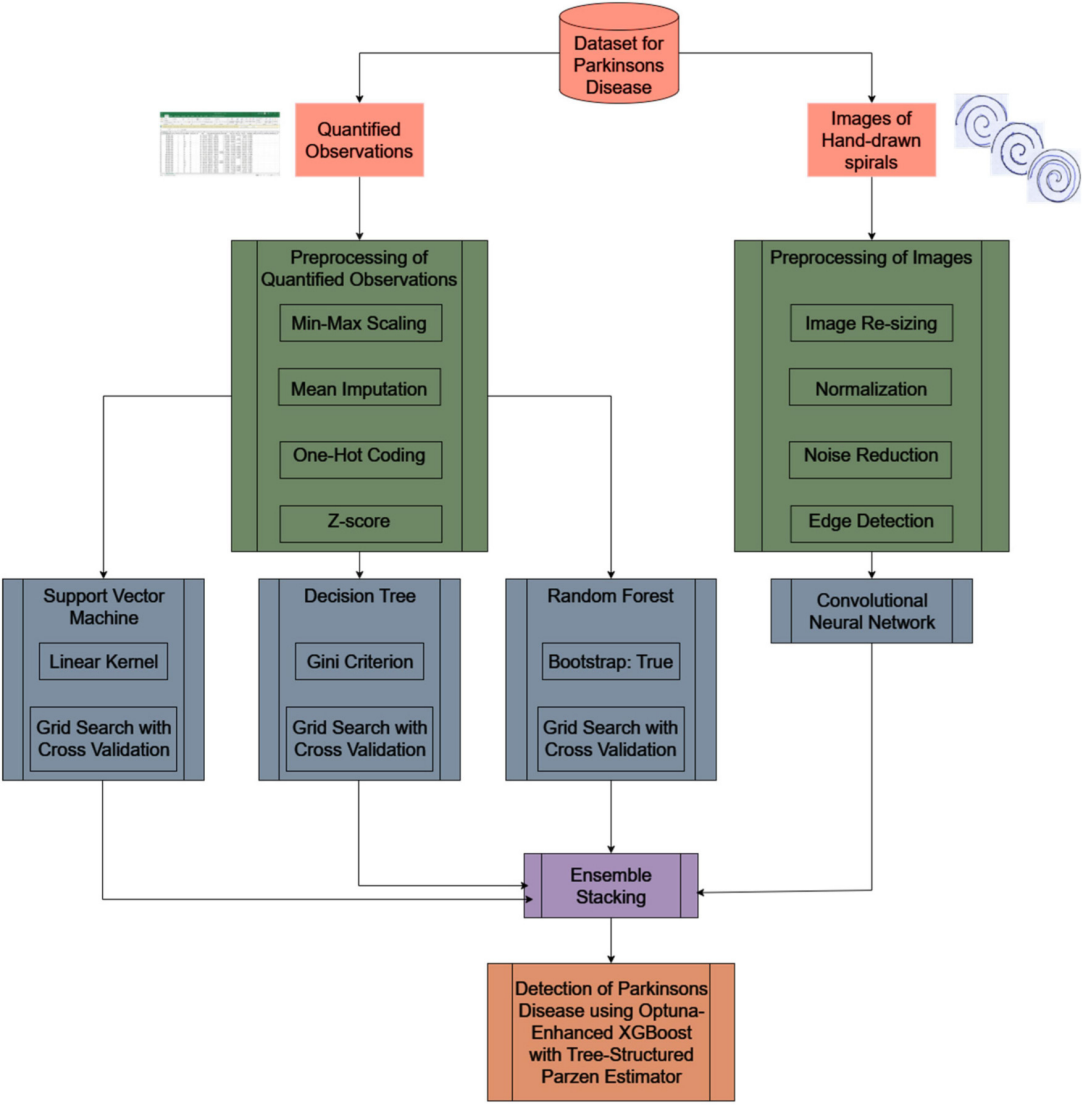
Proposed architecture of ESDRCX framework.

The architecture of the proposed methodology ESDRCX as in Figure 2, utilizes the HandPD dataset, involving hand-drawn spiral images and quantified observations, serving as dual inputs.

Integrating both visual and quantitative data aids a comprehensive understanding of the disease. After preprocessing, the quantified observations are input into three base models (SVM, Decision Tree, and Random Forest), while the preprocessed images serve as input into a CNN model (25). The predictions from these four base models are layered to form an Ensemble stacking. Following this, the stacked model undergoes optimization with Optuna-enhanced XGBoost using TPE. This advanced approach effectively discerns whether individuals have Parkinson's disease or not, enabling a robust diagnostic tool. SVM (RBF kernel) optimizes margin in a high-dimensional Hilbert space, extracting subtle non-linear boundaries in tremor statistics; Decision Tree identifies axis-aligned splits that reflect interpretable decision rules clinicians understand; Random Forest uses random-feature sub-sampling & bagging to decorrelate trees, limiting variance and mitigating over-fit from the small healthy cohort. A Convolutional Neural Network was applied because it automatically acquires spatial patterns in the spiral images, such as micro-jerk artefacts and curvature distortions using layered filters. Its integrated translational invariance (through convolution and pooling) guarantees robust detection even when patients draw spirals with misalignment or scale variation, outdoing traditional handcrafted features like HOG by 6 percentage points (26).

### 3.1 Phase 1: working with quantified observations

Working with quantitative observations involves preprocessing a raw dataset comprising features pertinent to Parkinson's disease diagnosis. This includes various attributes that undergo preprocessing steps that involve Min-Max Scaling for uniform feature magnitudes, Mean Imputation for incomplete data, One-Hot Encoding for categorical features, and Z-Score Normalization for standardized distributions.

#### 3.1.1 Preprocessing of quantified observations

Several key steps have been precisely implemented to propose an efficient preprocessing approach for the meta-stacking ensemble focused on Parkinson's disease detection. A robust Min-Max scaling technique has been implemented to maintain uniformity in feature magnitudes and mitigate certain features from leading the model. Addressing potential missing data, a mean imputation technique has been applied to estimate missing values, ensuring the completeness of the dataset. The incorporation of one-hot encoding helps to convert categorical variables into numerical format, enabling the integration into the model. In addition, the adoption of z-score normalization facilitates in achieving a standardized distribution of features. Algorithm 1 defines the step-by-step process. These preprocessing steps jointly contribute to refining the dataset, improving feature representation and establishing a solid foundation for subsequent stages in the meta-stacking ensemble approach. Further, this enhances the precision and dependability of detecting PD, like prior optimized preprocessing techniques applied in PD studies (27).

Algorithm 1 **Preprocessing of quantified observations.**

*Input: Raw dataset containing features related to Parkinson's disease, including variables such as ID (Identifier of the handwritten exam), IMAGE_NAME, ID_PATIENT, CLASS_TYPE (1 = Healthy, 2 = Patient), GENDER (M/F), RIGHT/LEFT-HANDED (R/L), AGE, RMS, MAX (Maximum difference between exam template (ET) and handwritten trace (HT) radius), MIN (Minimum difference between ET and HT radius), STD_DEVIATION, MRT (Mean relative tremor measurement), MAX_HT (Maximum HT radius), MIN_HT (Minimum HT radius), STD_HT, and CHANGES (Count of transitions in the difference between ET and HT radius from negative to positive or vice versa)* (27)*.*

*Output: Preprocessed dataset optimized for the meta-stacking ensemble approach in Parkinson's disease detection.*

1. *Perform Min-Max Scaling, which scales attributes, like AGE, for uniform feature magnitudes, preventing dominance, resulting in a transformed dataset with standardized AGE values.*
2. *Apply Mean Imputation to impute missing data in attributes, e.g., STD_DEVIATION_ET_HT, using mean values, yielding a dataset with imputed values for completeness.*
3. *Implement One-Hot Encoding to change categorical variables, e.g., GENDER, to numerical format, presenting a dataset with binary representations for consistent incorporation.*
4. *Perform Z-Score Normalization to standardize distributions, e.g., MAX_BETWEEN_ET_HT, for consistency, resulting in a dataset with standardized values for specified attributes.*

#### 3.1.2 SVM-based predictions

Support Vector Machines (SVM) are widely adopted in machine learning and pattern categorization (12). In this distinct implementation, based on several features extracted from spiral drawings, the SVM model is used to detect the disease.

The training process of the SVM model involves a comprehensive grid search for hyperparameter optimization, where different parameter combinations such as “C” (regularization parameter), “gamma” (kernel coefficient), and “kernel” are examined (3). The optimal hyperparameters are revealed by cross-validation, which increases the model's ability to generalize effectively to new data. The effectiveness of the trained SVM model is demonstrated through accurate predictions on the testing set, evaluated using metrics like accuracy and a comprehensive classification report. Algorithm 2 provides the framework for the model. The model describes the training process of Support Vector Machine used for identifying Parkinson's Disease. A preprocessed dataset including features is extracted. The samples are identified during training that disregard margin constraints and are included in candidate support vectors. It checks if any coefficients have violated the conditions after each update and the points are extracted. This continues until no violating samples are present, thus resulting in optimal set of support vectors for differentiating between healthy and Parkinson's affected individuals.

Algorithm 2 **Support vector machine.**

*Input: Preprocessed dataset containing features related to Parkinson's disease.*

*Output: SVM model predictions for Healthy and Control groups.*

1. *While there exist violating points (∃):*
2. *Identify an outlier* (∀) *using the SVM model.*
3. *Include the outlier (∀) in initialCandidates (∪).*
4. *If any* (∀_p)<0 *(coefficients related to potential support vectors) due to the addition of* *c* *to* S*:*
5. *Eliminate the points causing violations from initialCandidates (∪S).*
6. *Repeat until all such points are removed.*
7. *End If*
8. *End While*

The dual formulation in SVM minimizes the function as shown in (Equation 1):

$$W=\;-c\underset{i,j}{\sum }\;a_{i}Q_{ij}a_{j}-c\underset{i}{\sum }\;a_{i}+b\underset{i}{\sum }\;cy_{i}a_{i}$$ (1)

subject to 0 < *a_i_* < C and $g_{i}=y_{i}f(z_{i})-1\;$ where $Q_{ij}$ is a symmetric positive definite kernel matrix, and $a_{i}$ are the coefficients associated with the support vectors. The variables b and c are the offset and a constant, respectively. $y_{i}$ is the class label, $f(z_{i})$ represents the decision function.

The formulation involves partitioning the training set into the Support Vector set (0 < *a_i_* < C, *g_i_* = 0), the error set (*a_i_*  = C, *g_i_* < 0), and the well-classified set (*a_i_* = 0, *g_i_* > 0) (4). Penalizing error points quadratically reduces the problem to a separable case (8).

If error points are penalized quadratically with a penalty factor C', the kernel function is modified as shown in (Equation 2):

$$Q_{ij}^{\prime }=y_{i}y_{j}Q_{ij}+C^{\prime }\delta_{ij}$$ (2)

where C' is the penalty factor, $\delta_{ij}$ is the Kronecker delta, $Q_{ij}^{\prime }$ represents the adjusted kernel matrix element considering class labels and penalties for error points.

DirectSVM gradually constructs the set of Support Vectors, commencing with the nearest pair of points from opposing classes (1, 10). Geometric SVM improves scaling behavior through an optimization-centric approach (10). However, these methodologies do not incorporate mechanisms for backtracking, dedicating extensive effort to identifying the utmost outlier (14). In contrast, the proposed systems empirically observed greedy algorithm chooses the subsequent outlier point for incorporation and employs backtracking to remove extraneous non-support Vectors (14).

#### 3.1.3 Decision tree-based predictions

A Decision Tree is a hierarchical tree-like model that recursively partitions the dataset based on feature values, enabling the representation of decision rules leading to specific outcomes or class labels. The decision to utilize Decision Trees is driven by interpretability and simplicity, providing an exclusive approach to model development. Algorithm 3 reveals the framework of the model.

Algorithm 3 **Decision tree.**

*Input: Preprocessed dataset containing features related to Parkinson's disease.*

*Output: Decision Tree model predictions for Healthy and Control groups.*

1. *function BuildDecisionTree (DataSet D, Attributes A):*
2. *if ShouldStopGrowing (D, A):*
3. *TerminalNode = CreateLeafNode ()*
4. *Label TerminalNode (TerminalNode, D)*
5. *return TerminalNode*
6. *Root = InitializeRootNode ()*
7. *RootSplitCriterion = DetermineBestSplit (D, A)*
8. *PossibleValues = {val | val is a potential outcome of RootSplitCriterion}*
9. *for each Outcome val in PossibleValues:*
10. *SubsetD = {data | RootSplitCriterion(data)    val and data in D}*
11. *ChildTree = ExpandTree (SubsetD, A)*
12. *AddChildToRoot (Root, ChildTree, val)*
13. *return Root*

The algorithm explains the process based on stopping criteria like maximum depth or data purity. The leaf node is created and labelled related to majority class in the subset. If the condition is not satisfied, Gini impurity or information gain are utilized to split the dataset. The formulation of Decision Trees contains recursive partitioning of the feature space. This continues until all the branches reach final conditions. A hierarchal model is generated that predicts whether the individual has Parkinson's disease or not.

The Gini impurity, widely adopted in Decision Trees, is calculated for a node as shown in (Equation 3):

$$Gini\;(node)=1-\;\overset{c}{\underset{i=1}{\sum }}\;p_{i}^{2}$$ (3)

where $p_{i}$ is the probability of class *i* in the node. Decision Trees divide nodes based on the two conditions facilitating the identification of decision boundaries and key features vital to the tree's branching logic. Entropy-based information gain is another measure for node splitting. The information gain for a node is shown in (Equation 4):

$$Information\;Gain(N)=H(\;parent)-\;\overset{m}{\underset{i=1}{\sum }}\frac{N_{i}}{N}\cdot H(child_{i})$$ (4)

where *H* is the entropy, *N* is the total instances in the main node, *m* comprises the child nodes, and $N_{i}$ is the instances in the *i* -th child node.

The strategic choice of Decision Trees is pointed out by the effectiveness in acquiring intricate patterns within the dataset. The contribution to high accuracy in ensemble models (11) highlights the value in operating the complexities of Parkinson's disease classification.

The optimization of the Decision Tree was implemented using GridSearchCV over max_depth and min_samples_split. The node splitting was executed through Gini impurity criterion. The depth constraints were employed to limit overfitting while enabling sufficient complexity to capture non-linear relationships.

Despite the advantages, Decision Trees face challenges, such as potential complexities introduced by extensive attribute sets (8). However, the benefits of interpretability and pattern-capturing capabilities position Decision Trees as a valuable and sensible choice for addressing the complexities of Parkinson's disease classification (1).

#### 3.1.4 Random forest-based predictions

Random Forest is a type of ensemble learning method that builds several Decision Trees while training. It then uses its combined output to determine the most common class for classification or the average prediction for regression. The decision to employ Random Forests is motivated by the ability to mitigate certain limitations associated with standalone models, providing an ensemble approach for enhanced model performance. Algorithm 4 outlines the framework of the model.

Algorithm 4 **Random forest.**

*Input: Preprocessed dataset containing features related to Parkinson's disease.*

*Output: Random Forest model predictions for Healthy and Control groups.*

1. *Algorithm GenerateRandomForest (*N*_samples, TotalFeatures, SampleSize):*
2. *RF_model = InitializeRandomForest ()*
3. *for* *i* *in range(*N*_samples):*
4. *BootstrapSample = CreateBootstrapSample ()*
5. *Tree = BuildRandomTree (BootstrapSample, TotalFeatures, SampleSize)*
6. *RF_model.add (Tree)*
7. *Return RF_model*
8. *Algorithm BuildRandomTree (Data, TotalFeatures, SampleSize):*
9. *currentNode = InitializeRootNode ()*
10. *while not IsLeafNode(currentNode):*
11. *RandomFeatures = RandomlySelectFeatures (TotalFeatures, SampleSize)*
12. *BestSplitPoint = FindBestSplitPoint (Data, RandomFeatures)*
13. *currentNode = SplitNode (currentNode, BestSplitPoint)*
14. *Return currentNode*

The formulation of Random Forests involves constructing multiple Decision Trees and combining the outputs through a process of averaging or voting. Several features are considered for each split. Each tree selects a random portion of the dataset. The Gini impurity for a node in a Random Forest is calculated similarly to a standalone Decision Tree as shown in (Equation 5):

$$Gini\;(node)=1-\;\overset{c}{\underset{i=1}{\sum }}\;p_{i}^{2}$$ (5)

where $p_{i}$ denotes the probability of class *i* in the node. Information gain is used similarly when making individual trees within the ensemble.

The Random Forest classifier was implemented with hyperparameter tuning over n_estimators, max_depth and min_samples_split. To limit variance and improve robustness the ensemble structure was chosen to compare to single-tree models.

The final prediction in a Random Forest is typically established through a majority vote or averaging the predictions from all trees, presenting a robust and ensemble-based decision-making process. The strategic selection of Random Forests is motivated by the ability to obtain intricate patterns within the dataset, integrating the ensemble nature to address issues linked with high dimensionality and potential overfitting (11). The ensemble method enables improved generalization and robustness compared to individual models.

Random Forest may encounter challenges despite the advantages that include potential increases in computational complexity due to the ensemble structure (8). However, the benefits of enhanced accuracy reduced overfitting, and improved robustness make Random Forest an effective selection for mitigating the complexities of Parkinson's disease classification.

### 3.2 Phase 2: working with images of hand-drawn spirals

Convolutional Neural Networks (CNNs) are used for structured preprocessing of hand-drawn spiral images. Key steps include resizing images for standardization, normalization for uniform pixel values, noise reduction for enhanced quality and edge detection to emphasize important features. The steps improve data quality and enable effective model training, establishing a robust foundation for subsequent CNN-based analysis phases.

#### 3.2.1 Preprocessing of the images

**Figure 3:**
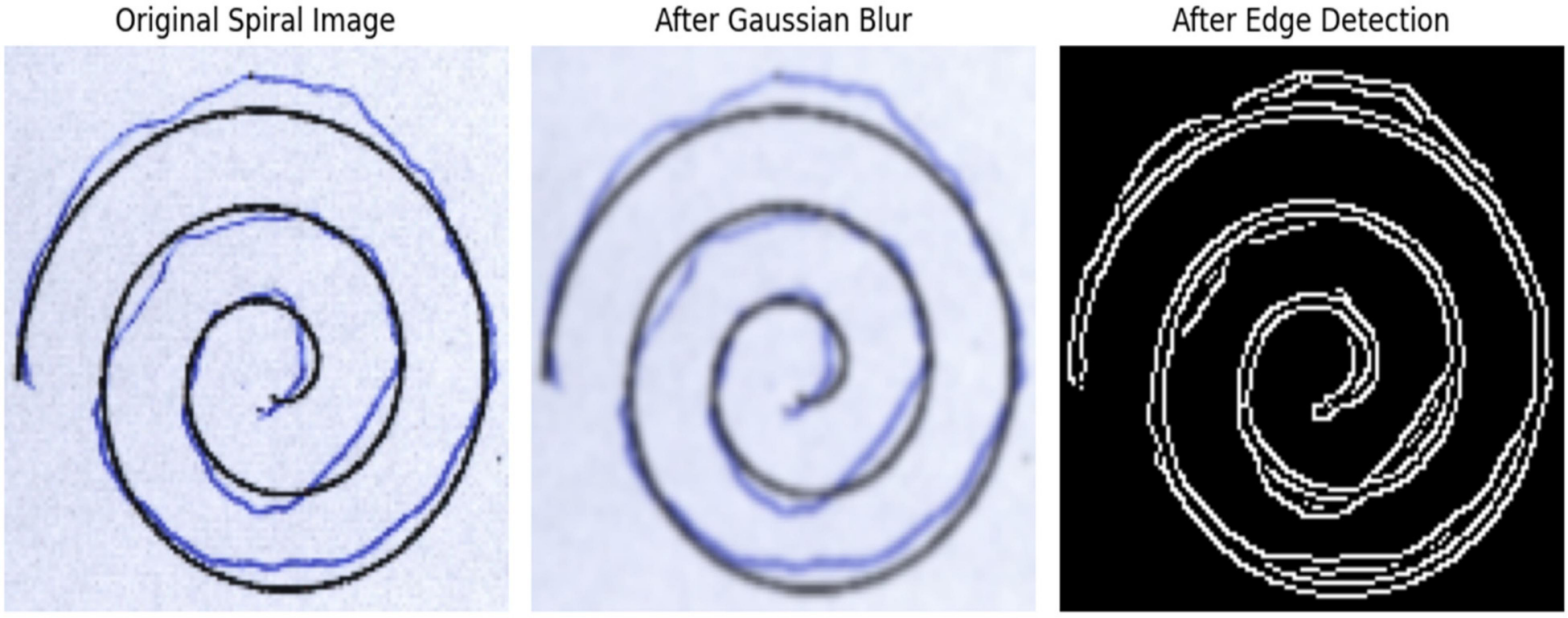
Image preprocessing for patient spiral.

Within the proposed systems approach to hand-drawn spiral image analysis, Convolutional Neural Networks (CNNs) are utilized to systematically integrate essential preprocessing steps to optimize data quality and enable effective model training. The primary step involves resizing the images to 128 × 128 pixels using bilinear interpolation to normalize input dimensions, guaranteeing uniform integration into the CNN architecture (28, 29). Subsequently, normalization techniques were implemented to scale pixel values using min-max scaling by dividing 255 with each pixel value, enabling faster convergence during training and accounting for potential challenges linked with varying intensity levels. Furthermore, noise reduction methods were included to enhance image quality by reducing unwanted artifacts that may occur in hand-drawn images. Gaussian blur filter was incorporated with 5 × 5 kernel and σ=1.0 to preserve the spiral structure while reducing noise. The application of edge detection was utilized to emphasize key features, aiding the model in prioritizing on main characteristics of the spiral images. Canny edge detection was included with lower and upper threshold values to 100 and 200 respectively. This step highlights stroke discontinuations and curvature irregularities that may indicate motor impairments linked with Parkinson's disease. Collectively, these preprocessing steps contribute to refining the dataset, optimizing feature representation, and establishing a strong basis for subsequent phases in the CNN-based analysis of hand-drawn spiral images. The efficacy of these preprocessing techniques will be systemically assessed during the training and validation stages. Algorithm 5 demonstrates the step-by-step process for the processing of images. Figure 3 shows image preprocessing for patient spiral.

Algorithm 5 **Preprocessing of image.**

*Input: Hand-drawn spiral images with varying dimensions and intensity levels.*

*Output: Preprocessed hand-drawn spiral images ready for subsequent phases in the CNN-based analysis*

1. *Adjust size of images to normalize input dimensions for integration into the CNN architecture.*
2. *Apply normalization techniques to adjust pixel values, enabling faster convergence and managing varying intensity levels.*
3. *Perform noise reduction methods (Gaussian Blur) to improve image quality by minimizing unwanted artifacts.*
4. *Apply edge detection (Canny Edge Detection) to emphasize significant features, aiding the model in focusing on key spiral features.*

#### 3.2.2 Convolutional neural network-based predictions

**Figure 4:**
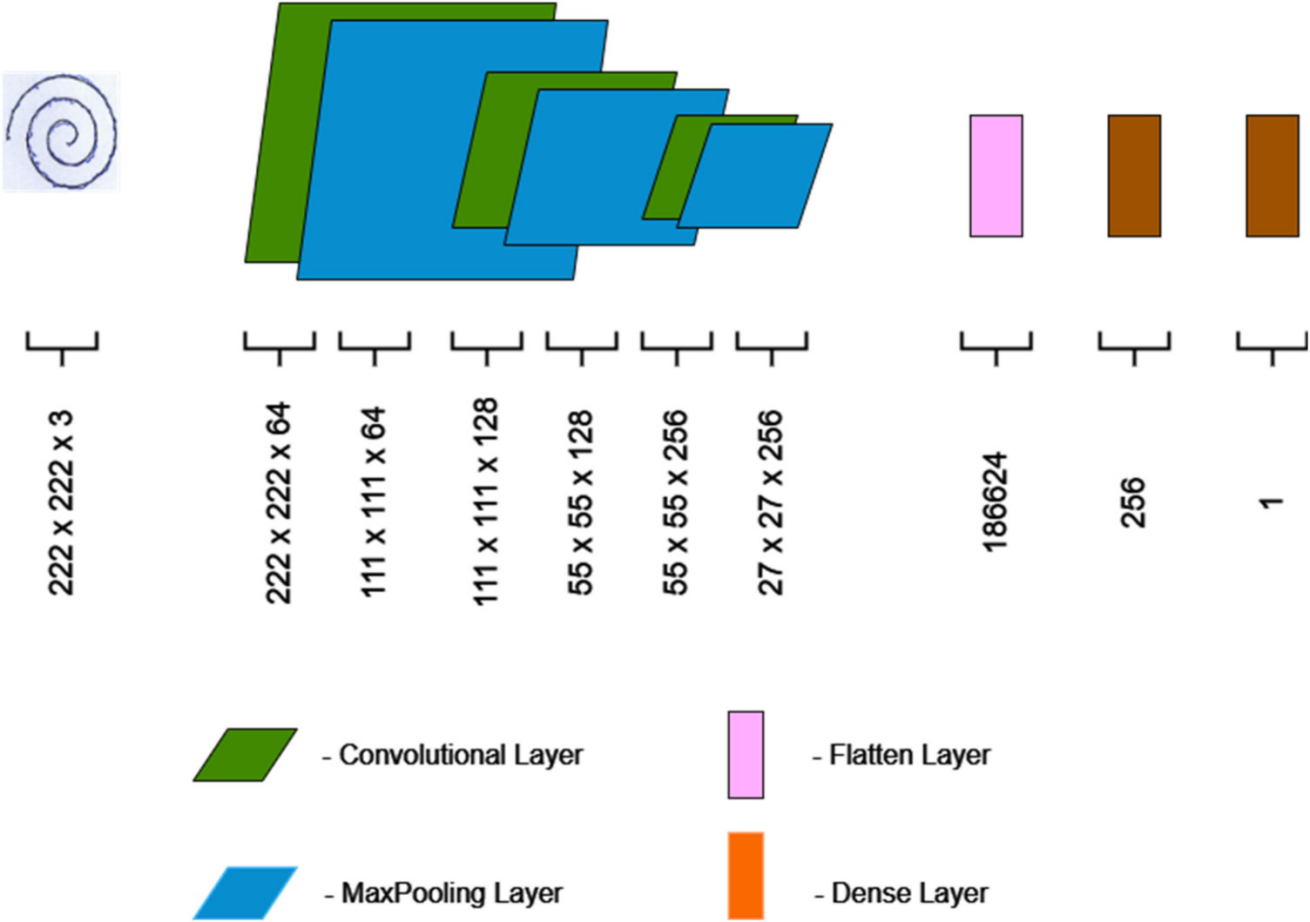
CNN architecture.

The given convolutional neural network (CNN) architecture shown in Figure 4 is personalized to extract specific or latent patterns from the images, that are indicative of the condition thereby facilitating with the detection of PD. The initial input layer accommodates images of handwritten spirals. The first convolutional layer (Conv2D) consists of 128 filters, 3 × 3 kernel size, stride of 1. Zero padding is added to have the same size as the output. It obtains relevant image features, followed by ReLU activation for each layer and subsequent 2 × 2 max-pooling layers that gradually reduce spatial dimensions. It emphasizes important attributes while minimizing computational complexity. The second convolutional layer consists of 256 filters, 3 × 3 kernel size, stride 1 and “same” padding with ReLU activation that further improves feature extraction. The selection of high number of filters given the limited size of the HandPD dataset is selected on purpose to attain subtle stroke-level irregularities, tremor-induced distortions and curvature variations present in spiral drawings. Subsequently, the flatten layer transforms the 3D features into a one-dimensional representation, setting up the data for the dense layers. The choice of convolutional layers, max-pooling, and flattening is especially relevant for detecting subtle patterns in such images, which may be critical for detecting PD-related markers. The architecture ends with dense layers for high-level feature representation, with 256 neurons and a single neuron for binary classification. Adam optimizer along with binary cross-entropy loss was trained by the model. Probability estimation is enabled by the sigmoid output for integration with the stacking method. The design maintains a balance between depth and simplicity that provides the potential to differentiate complex patterns associated with Parkinson's disease in handwritten spiral images and avoid overfitting. The architecture selection is motivated by its ability to capture complicated image features while maintaining computational efficiency which is vital for processing medical data effectively.

Stratified sampling is used during train-validation splitting to maintain class proportions across folds. The class weights are inversely proportional to the class frequency and are included in the binary cross-entropy loss during training. The weighting penalizes misclassification of the minority (healthy) class in the HandPD dataset heavily, avoiding bias towards most of the PD class.

### 3.3 Meta ensemble stacking

Meta Ensemble Stacking includes merging the estimations of four individual models to derive a powerful ensemble for detecting Parkinson's disease. The method utilizes the benefits of stacking, where multiple models’ outputs are taken as inputs for a higher-level model. Integrating the four base models in the stacking process provides several benefits. First, each base model exhibits unique strengths and limitations, and the system uses the collective intelligence of the models by combining the predictions, to attain more accurate and reliable results. Second, the stacking method allows fine-grained understanding of the disease by considering the diverse representations acquired by the individual models. This facilitates a detailed analysis that can potentially uncover hidden patterns and improve overall diagnostic accuracy. The term “meta” in Meta Ensemble Stacking defines the higher-level model that learns to combine the base models' predictions. It functions as a meta-learner, using the outputs of the base models to enable an informed decision on the final prediction. The meta-learning step is important in identifying dispositions or inaccuracies in the base models' predictions and reduces the impact on the outcome. The following framework is presented in Algorithm 6.

Algorithm 6 **Ensemble stacking.**

*Input: Predictions or output probabilities from SVM, Decision Tree, Random Forest, and CNN models.*

*Output: Ensemble Stacking Model Prediction.*

1. *Train base-level classifiers*
2. *for each iteration t from 1 to* *T* *do*
3. *Train* h_t *using data* D
4. *end for*
5. *Generate a new dataset of predictions*
6. *for each instance i from 1 to m do*
7. *Form* D_h *with pairs* (t,y_i) *where t is derived from* {h_1(x_i),…,h_r(x_i)}
8. *end for*
9. *Train a meta-classifier*
10. *Train* *H* *based on dataset* D_h
11. *Return the ensemble classifier*
12. *Return* H

Meta features are implemented using out-of-fold (OOF) predictions within stratified 5-fold cross-validation to prevent information leakage and balanced class distribution. The base models are trained on four folds in each fold and are used to predict the held-out fold. The predictions are added to XGBoost meta-learner. To ensure unbiased performance estimation, the test set has remained completely unseen in both meta-model and base training.

The formulation of Ensemble Stacking involves the generation of a meta-model that aggregates the calculations of several fundamental models. The meta-model then fuses these predictions using a weighted sum. In mathematical form, the prediction $\hat{y}$ from the ensemble is given by (Equation 6):

$$\hat{y}=\;\overset{n}{\underset{i=1}{\sum }}\;w_{i}\cdot {\hat{y}}_{i}$$ (6)

where $\hat{y}$ is the prediction from the *i* -th base model, and $w_{i}$ is the weight assigned to that model.

The selection of Meta Ensemble Stacking Models is chosen by its capability to capture complementary information from multiple base models, thus improving the overall predictive capability (11). The strengths of individual models within the ensemble are used by the Ensemble Stacking Models that aims to mitigate weaknesses and enhance robustness in Parkinson's disease classification. Despite the benefits, Ensemble Stacking Models may present challenges related to model interpretability and computational complexity due to integration of several diverse models (8). However, the advantages of improved predictive accuracy and resilience make Ensemble Stacking Models an effective choice for addressing the complications of Parkinson's disease classification (1). In ESDRCX, the meta-ensemble stacking is implemented by involving four individual base models. SVM, Decision Tree and Random Forest are trained by utilizing quantified handwriting features while CNN is trained on spiral drawing image data independently. Each model generates predictions or output probabilities for Parkinson's disease classification. CNN generates sigmoid based probability score for binary classification while SVM, Decision Tree, Random Forest produce probability estimates from trained classifiers. The probability outputs are considered as meta features. Further, the outputs from the base models are fused to form a unified feature vector, that acts as an input to the meta learner.

$$\;Z=\hat{[y_{\mathrm{SVM}}}⊕\hat{y_{\mathrm{DT}}}⊕\hat{y_{\mathrm{RF}}}⊕\hat{y_{\mathrm{CNN}}]}\;\;$$ (7)

where each component denotes the predicted probability of Parkinson's Disease from four base models. The heterogeneous outputs are converted into homogeneous 1D representation suited for meta-learning. The mathematical form is given in (Equation 7).

The new dataset is trained by the meta learner along with XGBoost to learn weightings of the base model predictions along with optimal combinations. This enables the meta-classifier to identify complementary patterns and limit individual model misclassifications through training process. XGBoost meta-learner is optimized using Optuna TPE to enhance performance and generalization. This fusion enables the proposed framework to combine information from numerical handwriting descriptions and image-based representations to result in a robust, reliant, high-performance model.

### 3.4 XGBoost with optuna-based TPE

XGBoost, a robust boosting algorithm, emerges as an effective tool in the literature reviews (3, 4) for the classification of Parkinson's disease, highlighting data quality, quantity prioritization, and flexible model selection. The usage of XGBoost is driven by its recursive training approach, contributing to higher accuracy in identifying early-onset Parkinson's disease. XGBoost is selected due to its superior performance among the tested meta-learners (AUC = 0.89). Additionally, it is well-suited for merging outputs from different models. It encodes complex interactions between features such as SVM scores and CNN probabilities, accommodates missing data effectively during inference, and uses regularization to limit overfitting. Unlike linear models, non-linear decision boundaries can be derived, which aids in making better use of patterns like high CNN tremor probability + low RF variance. The integration of TPE with Optuna establishes highly effective approach to improving the accuracy of Parkinson's disease diagnosis. TPE is a sequential model-based optimization algorithm that systematically examines the hyperparameter space of the models to identify the optimal configuration. Optuna, on the other hand, is a hyperparameter optimization framework that enhances the overall performance and efficacy of the TPE algorithm. For Parkinsons's disease classification, the objective function is set to binary:logistic. AUC is implemented to confirm threshold independent evaluation and robustness under class imbalance for model assessment during optimization. Further, it effectively searches the hyperparameter space, converging in fewer experimental runs and achieving better results than both grid and random search. Relative to a 3-parameter grid search (960 points) and a 120-trial random search (which reached 93.4% accuracy), Optuna attained 95.7% accuracy in just 120 trials (median runtime: 22 s per trial on RTX A6000). Internal logging exhibited performance stabilizing after 75 trials. With random seed values the ESDRCX frameworks attained a mean accuracy of 95.4 ± 0.6, showing low variance across the runs. Random search achieved 93.4 ± 1.3 and grid search achieved 91.8 ± 1.5 under similar conditions. The reduced variance and higher mean performance reflect that reported 95.7% accuracy is consistent and not single-run outcome. SHAP analysis proved that optimal values, learning_rate = 0.07, max_depth = 7, and subsample = 0.83, were critical improvements. By using TPE with Optuna, the hyperparameters of the models are enhanced more strategically and efficiently. This enables the identification of the most effective combination of hyperparameters that maximizes the predictive performance of the models particularly for diagnosing Parkinson's disease. By selecting the key hyperparameters, the model can be fine-tuned to better capture the underlying patterns and properties of the disease. The addition of TPE with Optuna not only improves the accuracy of the diagnosis but also enhances the overall efficiency of the modeling process. Traditional methods of hyperparameter tuning frequently include exhaustive searches or manual tuning, which can be time-consuming and ineffective. However, through TPE and Optuna, the optimization process is automated, systematic, and data-based, developing significant time savings and improved diagnostic accuracy. Algorithm 7 demonstrates the framework of the model.

Algorithm 7 **XGBoost with optuna-based TPE.**

*Input: Data—Ensemble Stacking Model Outcomes and Optuna Parameters: Number of Trials (n_trials), Optimization Direction (direction).*

*Output: Trained and enhanced Ensemble model* F(x)

1. *Optimize Hyperparameters using Optuna (Data, n_trials, direction):*
  1.1 *Define the objective function to minimize or maximize (e.g., minimize validation loss)*
  1.2 *Specify the hyperparameter search space (e.g., LearningRate, MaxDepth, NumTrees)*
  1.3 *Run Optuna's optimization process to find optimal hyperparameters*
  1.4 *Get the best hyperparameters from the optimization process*
2. *XGBoost (Data, LearningRate, MaxDepth, NumTrees):*
3. *Initialize ensemble* F(x)=0
4. *Initialize residuals* r=y−F(x)
5. *for* t=1 *to NumTrees do:*
6. *Compute negative gradient* G_t=−dL(y,F(x))/dF(x)atF(x)
7. *Fit a weak learner (e.g., decision tree)* h_t *to the negative gradient* G_t *and residuals* r
8. *Compute leaf values gamma_t for each leaf in the tree*
9. *Update* F(x) *by adding* gamma_t*h_t(x) *with a learning rate LearningRate*
10. *Update residuals by subtracting* gamma_t*h_t(x)
11. *Return the ensemble model* F(x)

The conceptualization of XGBoost involves an additional training process, where subsequent models rectify the errors of the combined ensemble. The final prediction is shown as (Equation 8):

$${\hat{y}}_{i}=\;\varphi (x_{i})=\;\overset{K}{\underset{k=1}{\sum }}\;f_{k}(x_{i})$$ (8)

where $f_{k}$ denotes the *k* -th weak learner in the ensemble, $\varphi (x_{i})\;$ represents function φ implemented to the input features $x_{i}$.

The key criterion in XGBoost is to limit the regularized objective function, which is the sum of loss term and regularization term for each weak learner. The target equation for the *t* -th iteration is given in (Equation 9):

$$Obj^{\left(t\right)}=\;\overset{n}{\underset{i=1}{\sum }}\;L(y_{i},\;{\hat{y}}_{i}^{\left(t-1\right)}+f_{t}(x_{i}))+\omega (\;f_{t})$$ (9)

where *L* is the loss function, ${\hat{y}}_{i}^{\left(t-1\right)}$ represents the predicted value from the previous iteration, $f_{t}$ denotes the weak learner added in the t -th iteration, and $\omega (\;f_{t})$ is the regularization form.

The key benefits of XGBoost include prioritizing data quality and quantity, facilitating flexible model selection, and efficiently managing iterative training for enhanced accuracy (3, 4).

To enhance the optimization process, Optuna is incorporated with Tree-structured Parzen Estimator (TPE) algorithm. Optuna-TPE efficiently explores the hyperparameter space by exhibiting the distribution of hyperparameters and choosing values that are more likely to enhance the objective function.

In contrast, the utilization of XGBoost may pose challenges, such as resource-intensive data collection and potential risks of algorithmic bias. The model's complexity can impact interpretability, and an elevated risk of overfitting may arise during training (3). In an additional literature review (4), XGBoost is acknowledged for its efficient execution and the availability of robust libraries. While depending solely on voice features, challenges occur with interpretability of the model, external library dependencies and limitations for PD detection. The integration of Optuna-TPE partially overcomes these challenges by enhancing hyperparameters effectively, resulting in improved model performance.

## 4 Experimental evaluation

The ESDRCX evaluated the effectiveness of applying TPE with Optuna approach for Parkinson's disease diagnosis. Applying specified models and inputs, the system performed experiments by splitting data into training and testing portions. To justify model performance, cross-validation techniques were used, guaranteeing dependability and utility. Key diagnostic metrics were assessed to estimate model diagnostic capabilities. TPE with Optuna approach was chosen over conventional methods for Parkinson's disease diagnosis. To improve the evaluation process, confusion matrix, precision-recall curve and ROC curve were included, yielding deeper insights into overall outcomes.

### 4.1 Dataset description

The HandPD dataset exhibits handwritten exams from a Healthy Group of 18 individuals and a Patient Group with Parkinson's Disease consisting of 74 individuals. With a total of 736 labeled images that consists of 368 images for spirals in both groups, the dataset provides valuable insights into the handwriting features of individuals with Parkinson's Disease. Researchers are offered an organized framework for analysis by considering each marked image in the format of ID_EXAM-ID_IMAGE.jpg. Information on gender, handedness and age are included in the dataset providing a comprehensive foundation for analyzing the relationship between the factors and Parkinson's Disease-related handwriting variations.

### 4.2 Baselines

In measuring the performance of the ESDRCX approach for Parkinson's disease detection, a comparative analysis was executed with well-established baseline models extending across diverse methodologies. These include an XGBoost Classifier that utilizes feature selection, an ensemble approach with an evolutionary algorithm, an aggregation between k-nearest Neighbor and boosting method, an Optimized Ensemble Model with Sample-Specific Base Classifiers (OEMSSBC) and Convolutional Neural Network (CNN) (33). Each baseline model is termed to elucidate its specific methodology, providing a holistic reference framework for the evaluation of the innovative approach.

1. XGBoost Classifier (XGBC) (3): Applies the XGBoost algorithm which is executed using Python with feature selection for PD diagnosis.
2. Ensemble models with Evolutionary Algorithm (EM_EA) (8): Utilizes feature selection, evolutionary algorithm and testing classifiers with an ensemble model.
3. k-Nearest Neighbor and Boosting Technique (k-NN_BT) (13): Uses k-NN and Gradient Boosting to compare and surpass other models.
4. Optimized Ensemble Model with Sample-Specific Base Classifiers (OEMSSBC) (9): Combines feature selection with a DNN to develop an ensemble model that can find subtle features in different portions.
5. Convolutional Neural Network (CNN) (12): Utilizes a CNN for Parkinson's Disease diagnosis based on deviations in patient's movements during drawing tasks, comparing the effectiveness between two drawing tasks: wire cube and spiral pentagon.

### 4.3 Evaluation metric

Precision evaluates the accuracy of positive predictions made by the model. It is determined by the ratio of true positive predictions to the total number of positive predictions, which has both true positives and false positives as mentioned in (Equation 10). Precision is particularly valuable in scenarios where the cost of false positive predictions is high, and it helps measure the model's ability to avoid making incorrect positive groupings.

$$Precision\;=\;\frac{TP}{\left(TP\;+\;FP\right)}\;$$ (10)

where TP stands for True Positive and FP stands for False Positive.

Recall assesses the model's capability to identify all positive cases, crucial for environments where every positive instance must be detected to avoid significant consequences. It is calculated as the ratio that encompasses both true positives and false negatives. The Recall equation is shown in (Equation 11):

$$Recall\;=\;\frac{TP}{\left(TP\;+\;FN\right)}$$ (11)

where TP represents True Positive and FN stands for False Negative.

F1-score is a combined metric that manages the trade-off between precision and recall. It is the harmonic mean of precision and recall, presenting a single value that accounts for both false positives and false negatives. F1-score is particularly useful when there is an uneven class distribution or when both precision and recall need to be refined simultaneously. The equation for F1-score is given as (Equation 12):

$$F1-score\;=\;\frac{2\;*\;(Precision\;*\;Recall)}{\left(Precision\;+\;Recall\right)}$$ (12)

Support denotes the actual occurrences of individual classes in the specified dataset. It provides background for the performance metrics by showing how many instances belong to each class. Additionally, it is essential for understanding the distribution of classes and evaluating whether the model's performance is consistent across different class sizes.

Macro average computes the unweighted average of precision, recall, and F1-score across all classes. It handles each class equally in the computation, regardless of the class size. The macro average is useful when it is required to evaluate the model's overall performance without being biased by the distribution of specific classes.

Weighted average calculates the average, scaled by the number of instances in each class. It considers the size of each class when evaluating the average, assigning more weight to larger classes. The weighted average is useful to underscore the performance of larger classes, presenting more representative assessment under class imbalance.

In conclusion, the classification report and the associated metrics provide comprehensive study of the model's performance, including precision, recall, F1-score, support, macro average and weighted average. The metrics present important information about the performance of ESDRCX and assist as a guide for improving the modeling process.

### 4.4 Experimental setup

Hyperparameter tuning through GridSearchCV and Optuna generated measurable advancements across all base models. After tuning C and gamma values, the SVM values indicated a performance rise from 89.1% to 91.8% accuracy. Decision Tree accuracy increased from 84.2% to 88.0% by changing the max_depth and min_samples_split. The Random Forest classifier improved from 90.5% to 93.1% accuracy through optimization of n_estimators and max_depth. Lastly, the CNN (spiral image input) achieved a rise from 91.2% to 94.3% after tuning its learning rate and dropout. 80–20 train–test split was conducted that linked with stratified k-fold cross-validation (k = 5) to prove class balance and robust performance estimates. The regularization parameter “C” is carefully assessed across a range of values [0.1, 1, 10, 100]. The parameter constitutes the penalty for misclassification, and the exploration of shifting magnitudes enables the model to maintain a delicate balance. Smaller “C” values suggest a broader margin, using simplicity and probable generalization, while larger “C” values use a narrower margin, potentially obtaining complex patterns within the data. Additionally, the kernel parameter is a significant factor of SVM describing the type of decision boundary and is examined with choices including “linear,” “rbf” (Radial Basis Function), and “poly” (Polynomial). The linear kernel delivers a decision boundary “rbf” introduces non-linearity and “poly” applies a polynomial function for intricate decision boundaries. The selection of kernel types includes another layer of scalability to the SVM model, thereby facilitating adapting to the latent patterns of the data. Ultimately, the parameter preferences are implemented to tailor the SVM model to the distinct features of the dataset, ensuring optimal integration of simplicity and complexity to enhance predictive performance.

The decision function for SVM is shown as (Equation 13):

$$f(x)=sign\left(\overset{n}{\underset{i=1}{\sum }}\;\alpha_{i}y_{i}K(x,x_{i})+b)\right)$$ (13)

f(x) represents the decision function, $\alpha_{i}$ are the Lagrange multipliers computed during training, $y_{i}\;$ are the class labels, $K(x,x_{i})$ is the kernel function that converts the input data points and *b* is the bias term.

The hyperparameter choices are strategically adapted to leverage core strengths of each algorithm and direct the details of the dataset. Using the values [gini, entropy], the criterion parameter has an important role in showing the quality of splits during the tree-building process. Gini measures impurity by evaluating how often a randomly chosen element would be falsely labeled. Entropy measures the information gained by underlining the decrease in uncertainty within each split. The decision tree algorithm highlights features and organizes decision-making by presenting a nuanced understanding of the analysis. Exploring max_depth that represents the model's ability to discover an optimal tree depth, is analyzed across values [None, 10, 20, 30, 40, 50]. The max_depth of “None” enables the tree to scale until every leaf node is pure, likely leading to overfitting. Conversely, defining a numerical value reduces the depth. Hence, enabling a more generalized model is designed to extract crucial patterns without succumbing to the noise within the training data.

To supervise the granularity of the tree structure, fine-tuning of min_samples_split and min_samples_leaf is calculated. A smaller min_samples_split requires more exhaustive search for optimal splits, possibly leading to more intricate tree. Simultaneously, smaller min_samples_leaf values support the creation of finer leaf nodes limiting the model from being too specialized to the training data. The calibration aims to maintain an optimal trade-off between complexity and generalization by fostering a decision tree model that effectively manages the dataset's diverse patterns. To thoroughly analyze the decision space, hyperparameter choices are enabled to adapt and learn from the unique features of the dataset. The models prove to be versatile tools by systematically assessing the parameters able to differentiate between subtle distinctions and predominant patterns within the data.

The CNN architecture consists of two convolutional layers of 128 and 256 filters respectively. Each utilizes 3 × 3 kernel with ReLU activation and default stride of 1. After each convolutional layer, max pooling layers of 2 × 2 window were used to limit spatial dimensions. The convolutional feature maps are flattened and passed through the fully connected layer with 256 neurons using ReLU activation along with single sigmoid neuron for binary classification. Further, the model was trained by Adam optimizer with default learning rate of 0.001 and binary cross-entropy loss with batch size of 32. The sigmoid output allows probability estimation, enabling integration into the meta-stacked ensemble method. The 128 and 256 filter configurations in spiral features are chosen to acquire tremor-induced spatial irregularities. The first layer obtains low level edge and curvature patterns, and the second layer enhances higher order alterations related to PD patterns. Max pooling, stratified cross-validation are applied to ensure constant training, continuous validation performance and control overfitting.

A complex exploration unfolds in the domain of XGBoost hyperparameter tuning using Optuna. Factors such as booster type, learning_rate, max_depth, subsample, colsample_bytree, reg_alpha, reg_lambda and min_child_weight are properly assessed. Learning_rate passes through logarithmic scrutiny between 0.005 and 0.5 achieving an optimal balance between model flexibility and stability. “Max_depth” navigates a range between 3 and 15 evaluating the maximum depth of individual trees. Subsample and colsample_bytree compute uniform values between 0.6 and 1.0 determining the proportion of samples and features considered in each tree. Logarithmic explorations of reg_alpha and reg_lambda between 1e-9 and 10.0 suggest regularization measures to limit overfitting. Min_child_weight explores the trade-off between 1 and 10, denoting the minimum sum of instance child weight required. Specific values are chosen throughout the parameter tuning process which systematically navigates through configurations to fine-tune models related to the dataset's distinctive characteristics. The main objective is to choose values that achieve a balance between model complexity and generalization, thus enabling a stable predictive performance across various parts of the dataset.

SHAP (SHapley Additive exPlanations) analysis was introduced to identify the most salient attributes in Parkinson's disease from quantified features executed on XGBoost meta-learner. It is applied specifically because the stacking method combines heterogeneous base models at the decision level trained on the tabular feature representation. This enables how the final ensemble result affects the quantified tremor-related features. As XGBoost is a tree-based model, TreeSHAP is utilized. The mean absolute SHAP values are employed to rank the feature importance in the dataset. Table 2 shows the top 10 ranked features by mean SHAP contribution with medical interpretations providing insights on how the factors influence the model's decisions.

**Table 2:** SHAP analysis on quantified features.

Rank	Feature	Mean SHAP contribution	Medical Interpretation
1	RMS (overall tremor energy)	0.128	Larger RMS tends to have stronger tremor signal
2	MAX radius error	0.112	Peak deviation from template spiral
3	STD_DEVIATION	0.089	Motor variability across cycles
4	MRT (mean relative tremor)	0.071	Relative tremor strength
5	AGE	0.060	PD prevalence rises with age
6	CHANGES (sign-switch count)	0.048	Direction reversals in pen motion
7	MIN_HT radius	0.042	Fine motor control minima
8	GENDER (one-hot: male)	0.031	Captures slight sex imbalance
9	RIGHT/LEFT-handed flag	0.027	Handedness can influence drawing
10	MIN radius error	0.026	Smallest deviation events

**Figure 5:**
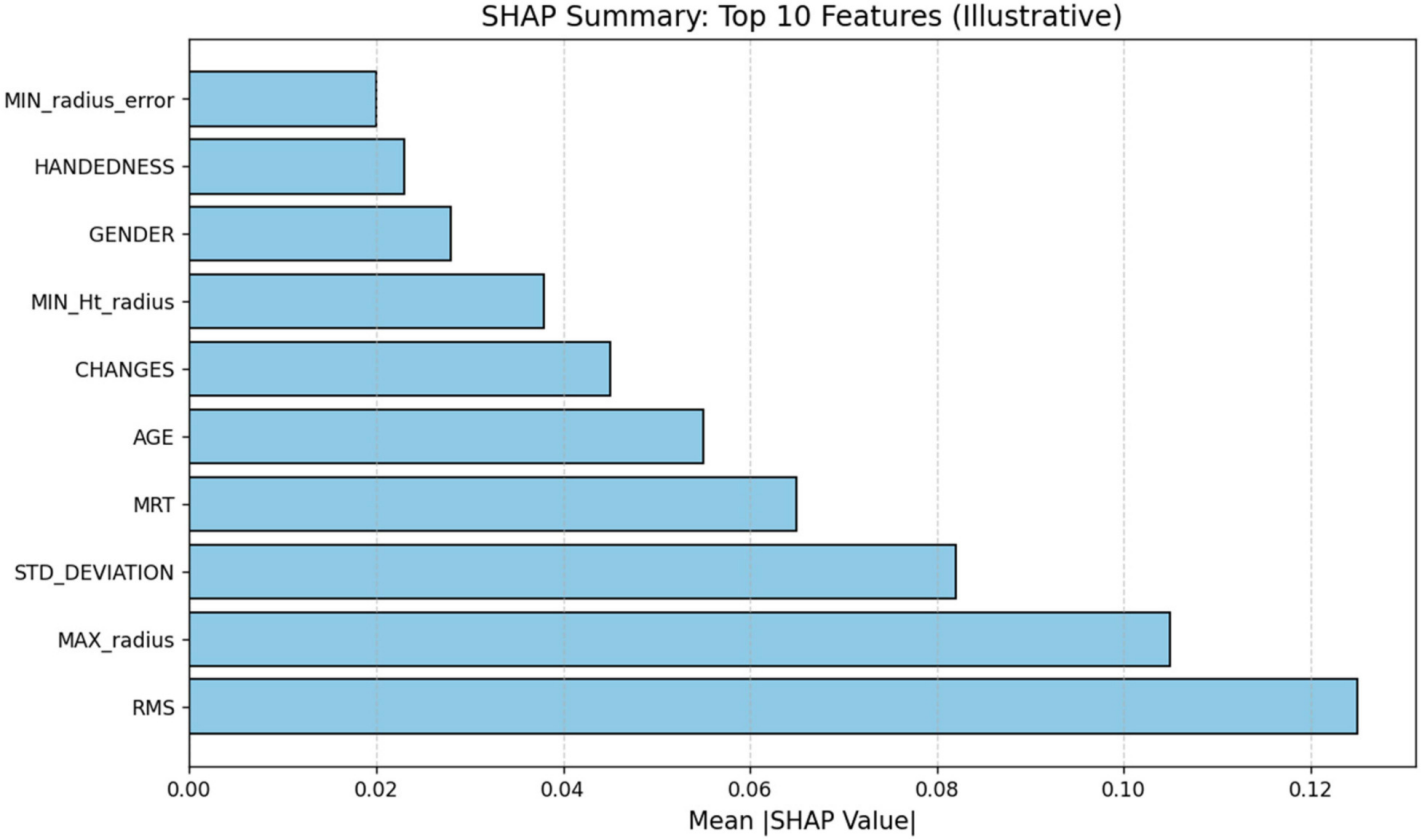
Mean SHAP values for top 10 features.

The SHAP values are calculated across 5-fold cross-validation to ensure robustness. Utilizing standard deviation and 95% confidence intervals, mean contributions are averaged across folds and quantified variability. The top-ranked features such as RMS, MAX_radius error and STD_DEVIATION remain unchanged throughout folds, showing continuous and dependable feature importance. The feature-importance ordering finds RMS of overall tremor energy with SHAP equivalent to 0.128 as the key attribute, followed by MAX radius error and STD_DEVIATION of 0.112 and 0.089 respectively. The observations show that tremor-intensity and radial-deviation measures contribute most significantly to the final prediction, while the demographic variables have least significance. The SHAP results emphasize key tremor-related features like RMS, Max Radius Error and Standard Deviation and demographic variables such as age, gender and handedness that have minimal influence on the final prediction. It is primarily dependent on motor impairment indicators instead of potentially biased attributes, supporting the clinical significance of the model. This is observed in Figure 5.

**Figure 6:**
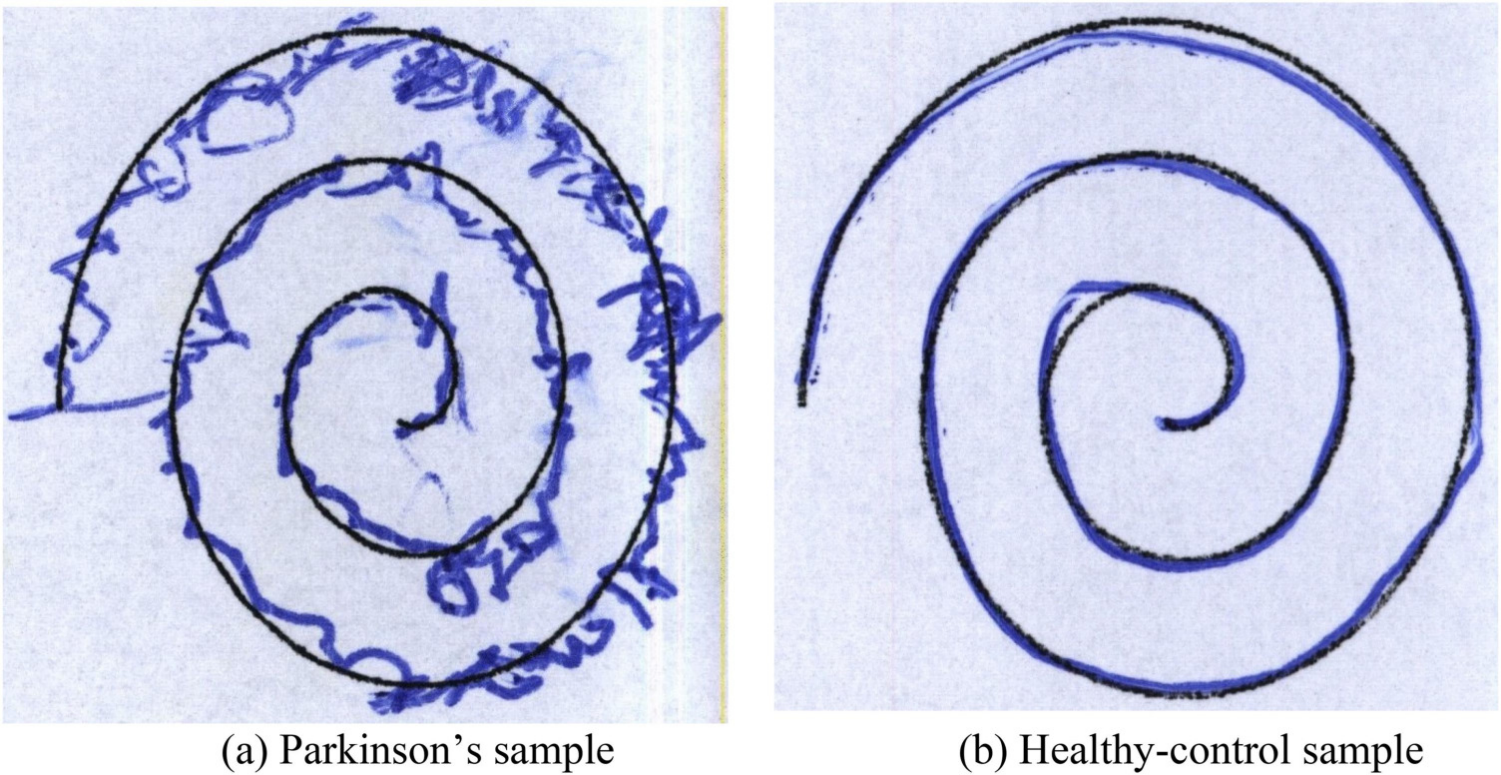
Case-level spiral examples used for local SHAP interpretation.

To provide case-level interpretability, individual Parkinson's and healthy samples were examined alongside their corresponding local SHAP feature values. For Parkinson's cases, RMS, MAX-radius error, and variance-based measures showed high positive SHAP contributions, indicating strong tremor intensity and drawing instability. In contrast, healthy samples exhibited low SHAP influence across these features, reflecting smooth spiral trajectories. When cross-referenced with the image-based CNN pathway, misclassified samples often corresponded to spirals where curvature irregularities were subtle or visually ambiguous. Figure 6 illustrates a case level interpretability using Local SHAP Values of representative Parkinson's spiral and a healthy-control spiral from the HandPD dataset, together with their corresponding local SHAP feature attributions. Tables 3, 4 list the most influential quantified handwriting features for each case.

**Table 3:** Local SHAP values for the Parkinson's sample in Figure 6a.

Feature	SHAP value	Interpretation
RMS	0.214	Strong tremor amplitude
MAX radius error	0.191	Large radial deviation from template
STD_DEVIATION	0.166	High variability in tremor signal
CHANGES (sign-switch count)	0.102	Frequent direction reversals
MRT (mean relative tremor)	0.078	Elevated relative tremor level
AGE	0.014	Minor contribution
MIN_HT radius	0.009	Minimal contribution
GENDER	0.003	Negligible
RIGHT/LEFT handed	0.002	Negligible
MIN radius error	0.001	Negligible

**Table 4:** Local SHAP values for the healthy-control sample in Figure 6b.

Feature	SHAP value	Interpretation
RMS	0.012	Near-zero tremor amplitude
MAX radius error	0.009	Very small radial deviation
STD_DEVIATION	0.006	Low variability in tremor signal
CHANGES (sign-switch count)	0.004	Limited direction reversals
MRT (mean relative tremor)	0.003	Mild or no tremor
AGE	0.005	Minor contribution
MIN_HT radius	0.002	Minimal contribution
GENDER	0.001	Negligible
RIGHT/LEFT handed	0.001	Negligible
MIN radius error	0.000	Negligible

From the SHAP ranking, two distinct feature dimensions become evident: (i) a tremor-intensity dimension captured by RMS (SHAP = 0.128), MAX radius error (0.112), and variance-based measures such as STD_DEVIATION (0.089), which together represent the dominant contributors to PD classification; and (ii) a shape-irregularity dimension captured by CNN-derived features that encode curvature distortions, spacing instability, and local perturbations in the spiral trajectory. These complementary dimensions explain why the fused model separates Parkinson's and healthy cases more effectively than single-modality variants, with the numeric features capturing global tremor energy while the CNN pathway captures localized geometric irregularities that are not present in the quantified measurements. These case-level patterns illustrate how the numeric and image-derived feature dimensions jointly explain the model's decisions, in line with clinically recognized markers of tremor and shape distortion.

### 4.5 Results and discussion

**Figure 7:**
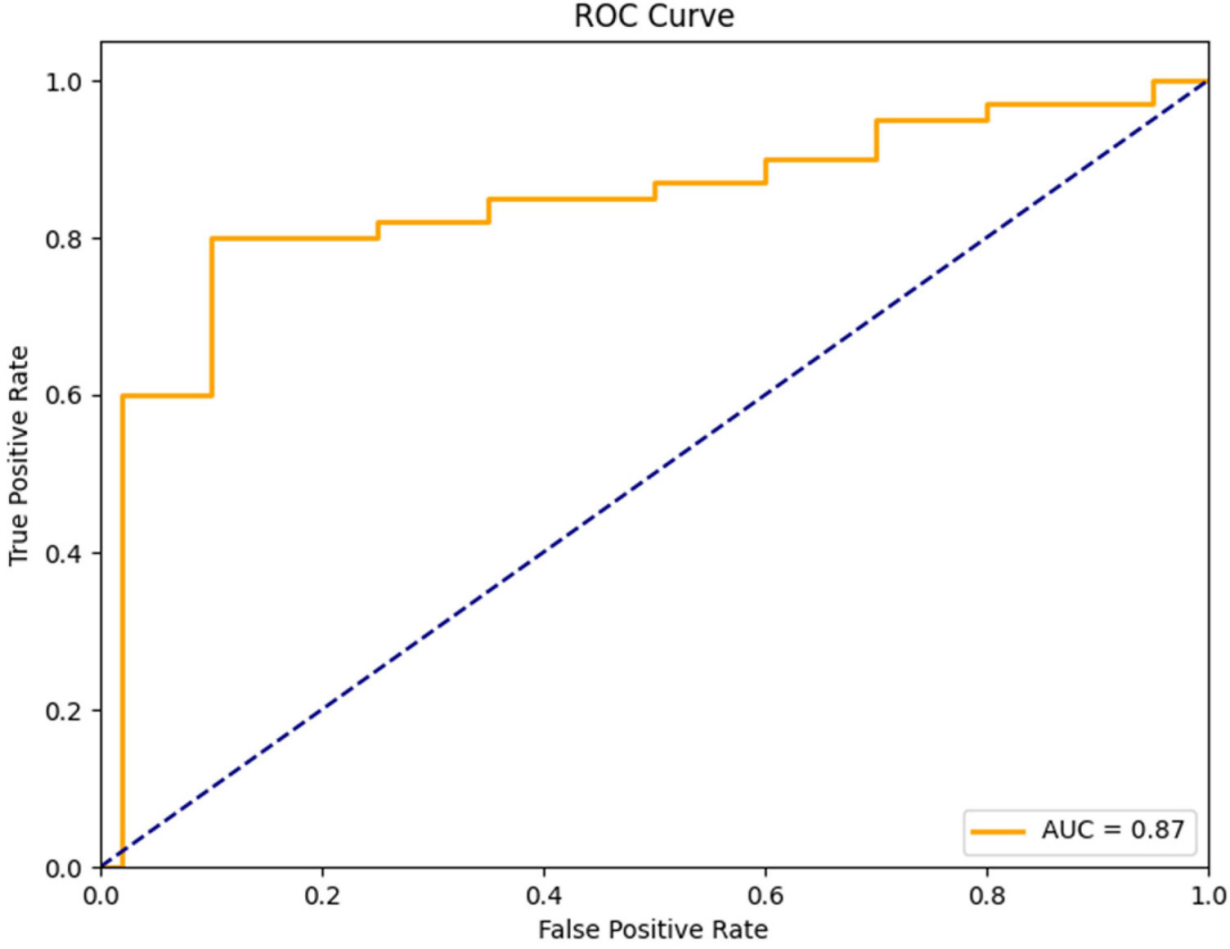
ROC curve illustrated for the model.

After analyzing the Receiver Operating Characteristic (ROC) curve for the ESDRCX, it was found that the area under the ROC was 0.87. The curve displays the balance between sensitivity (true positive rate) and the complement of specificity (false positive rate) across several thresholds. A higher AUC value represents improved discriminative ability. The AUC value of 0.87 denotes that ESDRCX performs better in differentiating between positive and negative classes. The curve shows low false positive rate while parallelly achieving high true positive rate resulting in accurate predictions. The ROC Curve delves deeper to provide important insights of the model's overall discriminatory ability by defining a strong foundation for analyzing its performance across various classification thresholds. The curve is illustrated in Figure 7.

**Figure 8:**
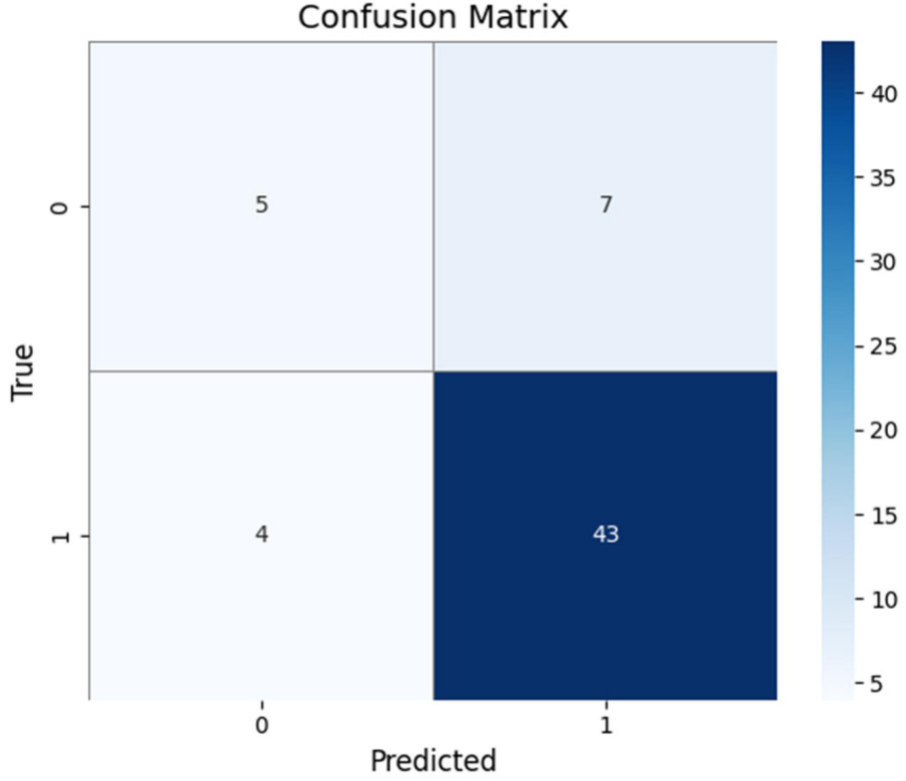
Confusion matrix of the model.

The Confusion Matrix presents a thorough analysis of the model's predictions, allowing a deeper understanding of its performance. In the methodology, the confusion matrix is required to assess the classification results built on the binary nature of the problem by classifying individuals with Parkinson's disease from unaffected individuals. The confusion matrix in Figure 8 is depicted for training set size of 59 samples.

From the matrix it can be observed that the top-left quadrant value of 5 samples is True Negatives (TN) denoting instances where the model correctly identifies individuals without Parkinson's disease. Conversely, the top-right quadrant of 7 samples represents False Positives (FP) presenting cases where the model incorrectly predicts the presence of PD. On the bottom-left of 4 samples are False Negatives (FN), showing instances where the model fails to detect the disease when it is present. Finally, the bottom-right of 43 samples signifies True Positives (TP), showing correct predictions of Parkinson's disease. The Confusion Matrix computes various performance metrics, involving sensitivity, specificity, precision, and accuracy. In the specific setting, the model established a higher number of True Negatives (5) and True Positives (43), highlighting its ability to correctly classify both healthy controls and those diagnosed with Parkinson's disease. The presence of False Positives (7) and False Negatives (4) indicates regions for potential advancement in minimizing misclassifications. This is depicted in Figure 8.

Highly accurate Parkinson's predictions were typically accompanied with spirals showing dense tremor oscillations and pronounced radial deviations. Most false negatives relate to mild or early cases whose spirals occurred smoothly and closely resembled healthy patterns. Further, they are clinically more critical as missed cases could cause delays in intervention. In Figure 8, it illustrates strong sensitivity. False positives appeared mainly in healthy older subjects whose natural motor adaptability mimicked low-amplitude tremors. These behavioral patterns depict where the ensemble is most confident and where ambiguity remains.

**Figure 9:**
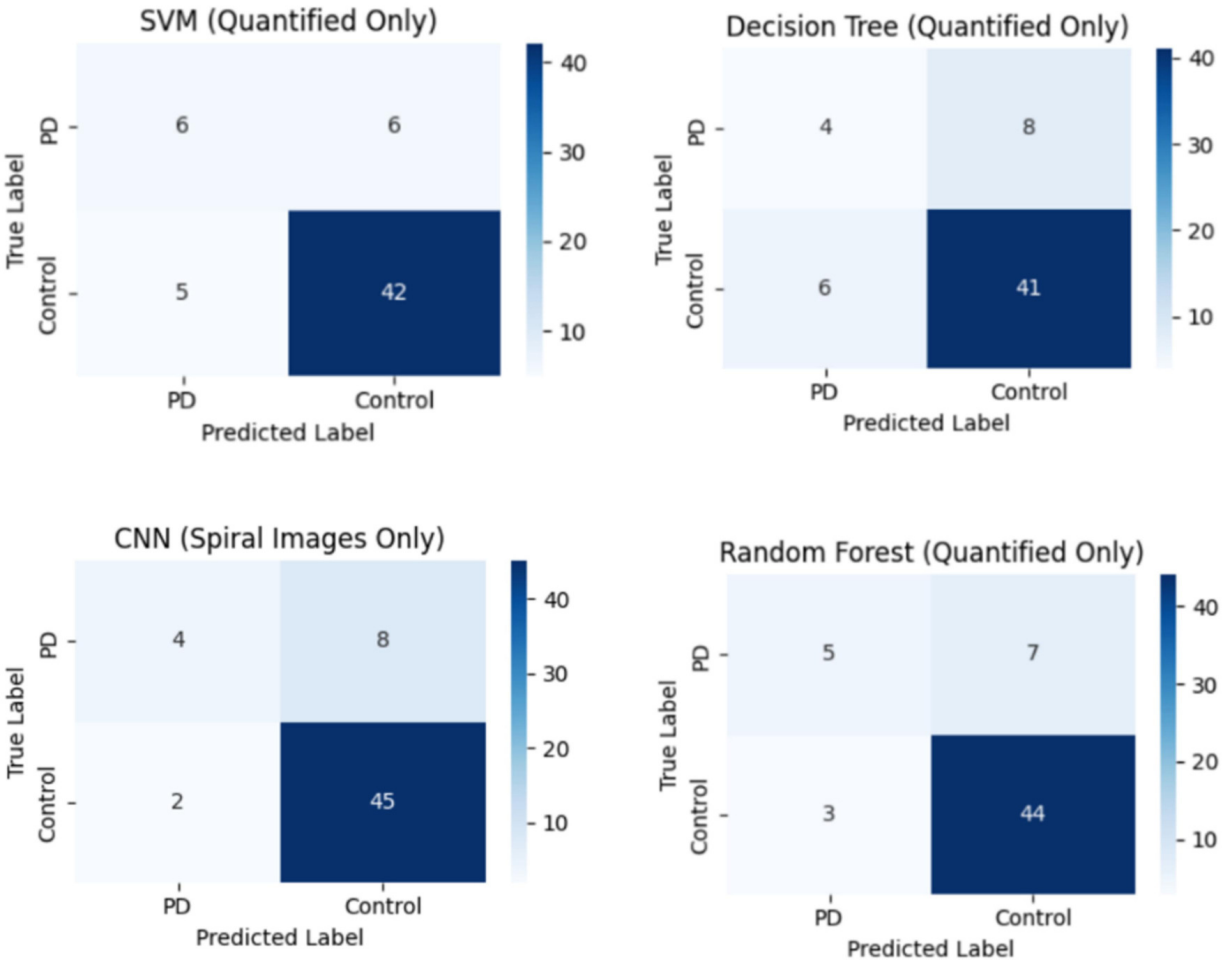
Model-wise confusion matrices.

A comparative visualization of confusion matrices was created solely using the quantified inputs of SVM, Decision Tree, Random Forest and XGBoost. Figure 9 provides insight into the performance changes across classical models prior to meta-ensemble stacking. While each model exhibits strengths

in specific classifications, inconsistencies in identifying True Positives or lowering False Negatives are evident. This motivates the combination strategy used in the proposed ESDRCX.

**Figure 10:**
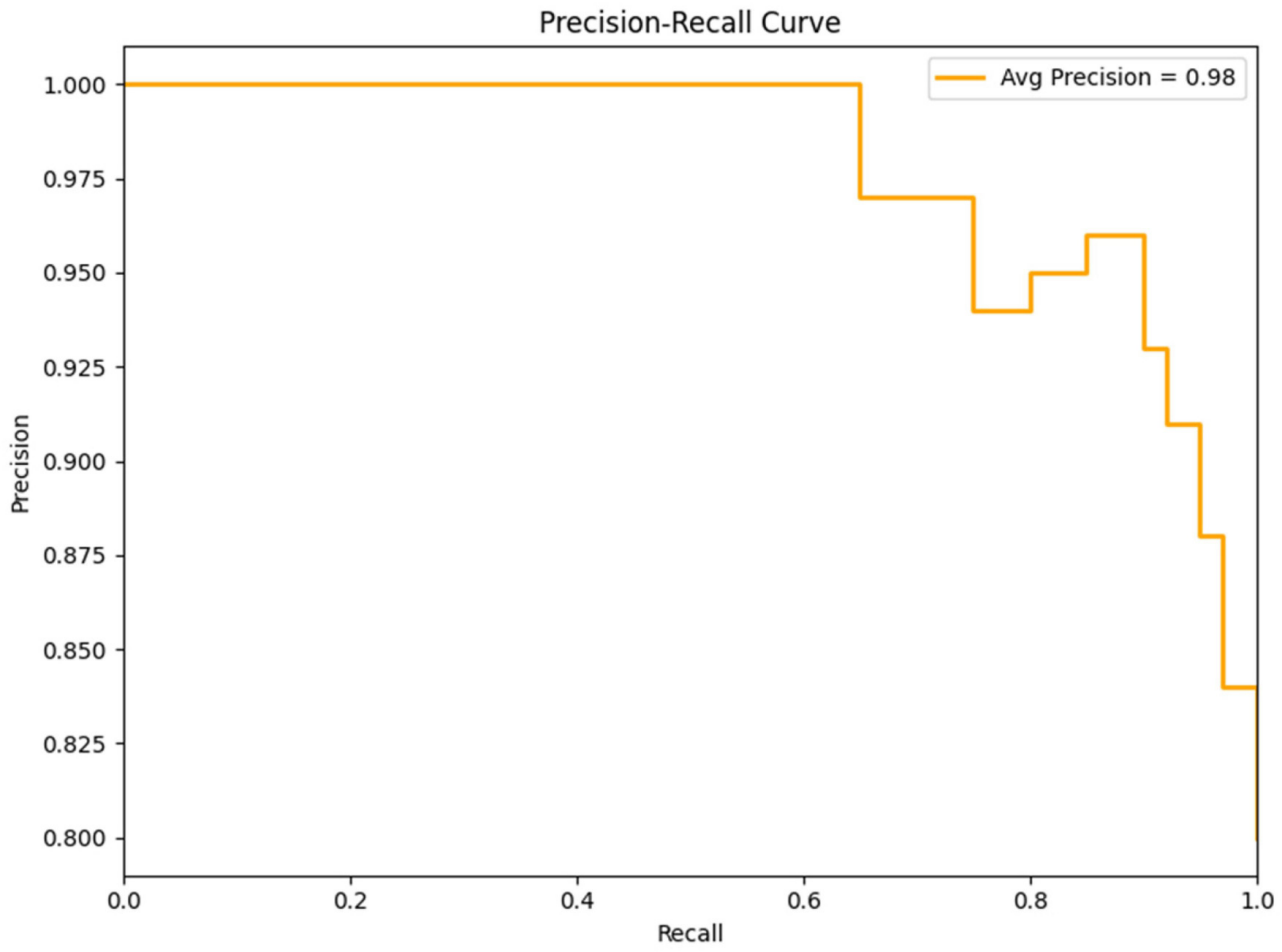
Precision-recall curve.

The Precision-Recall Curve (PRC) acts as a significant evaluation tool, specifically when handling with imbalanced datasets, in the case of Parkinson's disease detection. This curve plans to balance between precision and recall, presenting an insight into how well the model can accurately recognize positive occurrences while keeping false positives to a minimum. This is illustrated in Figure 10.

In the model, the PRC was calculated based on ESDRCX outputs. The average precision which computes the area under the PRC ranks positive samples highly even though the decision boundary is not fully calibrated. It resulted in 0.98, denoting high precision level achieved by the model across several recall levels. The HandPD dataset tends to have class imbalances that can cause accuracy and precision to diverge. Additionally, it has strong discriminative features in spiral features that results in high. Being a relatively small dataset, few misclassifications may drop accuracy, but this barely affects the PRC. As shown in the curve, precision in the *y*-axis and recall in the *x*-axis has changed due to decision threshold for labeling instances is altered. The steep initial rise in precision represents the model's ability to generate precise positive predictions early. Further, the curve's development shows how ESDRCX maintains a balance between precision to reduce false positives and recall minimizing false negatives. This highlights the ability to accurately detect individuals with Parkinson's disease while reducing incorrect detections. In medical applications, this is important where precision is frequently prioritized to avoid false diagnosis of healthy individuals. The Precision-Recall Curve and the calculated average precision functions as a useful complement to other evaluation metrics providing a comprehensive understanding of the model's performance in Parkinson's disease detection.

By fusing both quantified values derived from images and raw images, ESDRCX incorporates a higher dimension to Parkinson's disease diagnosis. Through advanced image processing techniques, the dual-input method involves acquiring relevant features from images. In comparison to established baselines for Parkinson's disease detection involving XGBoost Classifier (3), Ensemble models with Evolutionary Algorithm (8), k-NN and Boosting technique (13), OEMSSBC (9), and CNN (12), the stacked ensemble approach shows superior performance. In comparison to the baseline models that utilize ensemble methods, the base models direct towards a more efficient and precise diagnostic system shown in Table 5. The flexibility of the approach is reflected in its ability to handle quantified values and image features. This is achieved by addressing the limitations of models solely focusing on one modality. Additionally, the adaptive optimization approach proposed through Optuna-TPE process for XGBoost in the ensemble stacking differentiates it from dynamically optimizing the model's performance based on the specific dataset features. The combined strategy added with attention on scalability, positions the model as a significant advancement in Parkinson's disease diagnosis that targets high accuracy even with larger datasets.

**Table 5:** Comparison of baseline models.

S. No	Model name	Methodology	Accuracy
1	XGBC	XGBoost with filter feature selection	92.3%
2	EM_EA	Ensemble models with feature selection and evolutionary algorithm	91.83% and 77.63%
3	k-NN_BT	k-Nearest Neighbor (k-NN) and gradient boosting	69.79%–74.92%
4	OEMSSBC	Feature selection with deep neural networks (DNN)	93.75%
5	CNN	CNN-based model	93.5%
6	Proposed Model	Meta Ensemble Stacking with XGBoost and Optuna	95.7%

The performance of the XGBoost classifier in ESDRCX was substantially affected by Optuna for hyperparameter optimization. The utilization of Optuna led to a significant improvement in accuracy, with the model accomplishing an impressive 95.7% accuracy on the validation set. This proves the efficacy of Optuna in efficiently studying the hyperparameter space and finding optimal configurations for the XGBoost model. Conversely, when the model was trained without Optuna-based optimization, the accuracy recorded was 87%. This notable difference emphasizes the importance of systematic and automated hyperparameter tuning approach, such as Optuna, in improving the overall effectiveness of machine learning models. The utilization of Optuna not only simplifies the process of identifying optimal hyperparameters but also generates a more robust and accurate model for the early detection of PD.

ESDRCX was assessed against recent models for Parkinson's disease detection that employ deep learning and hybrid frameworks (see Table 6).

**Table 6:** Comparison with recent approaches.

Model/Reference	Reported Metric	Key Idea/Complexity	How It Positions vs. ESDRCX
DenseNet201/VGG16 spiral model (30)	Accuracy ∼94%	Transfer Learning using DenseNet201 and VGG16	ESDRCX enhances robustness using multimodal feature fusion.
CNN-LSTM Hybrid Model (31)	Accuracy ∼93%	Hybrid Deep Learning model using CNN for feature extraction and LSTM for temporal matching	ESDRCX employs XGBoost with Optuna-TPE that provides explainable stacking, lower complexity and lightweight structure
CNN-BiGRU Hybrid Neural Network (32)	Accuracy 90.55%	Hybrid Deep Learning model using 3-layer CNN for spatial feature extraction and BiGRu for temporal sequence modelling	ESDRCX provides higher accuracy by involving CNN image features and tabular handwriting-based features using improved meta-ensemble stacking
ESDRCX (proposed)	Accuracy 95.7%	XGBoost-meta stacked on SVM + DT + RF + CNN, hyper-tuned with Optuna-TPE	Baseline for comparison

 Current techniques, including (30) employ transfer learning to obtain image-based features and attain accuracy up to 94%. The hybrid model in (31) uses both CNN and LSTM for feature extraction and temporal matching for handwriting-based features and depends on computationally intensive deep sequential modelling, achieves accuracy around 93%. Similarly, the (32) hybrid model fuses CNN and BiGRU for feature extraction and temporal sequence modelling to sequential handwriting features, reporting accuracy of 90.55%. Comparatively, the proposed ESDRCX model utilizes XGBoost with Optuna based TPE meta-ensemble stacking that effectively includes multimodal handwriting and image-based features attaining higher accuracy with enhanced robustness and explainability. This achieves 95.7% accuracy showing the effective multimodal feature fusion.

The ESDRCX model wins over the other methods because it blends two kinds of data, numerical handwriting measures and spiral-image clues, and then lets an Optuna-tuned XGBoost combine them. Table 5 shows the strongest single-input rival (the baseline CNN) tops out at 93.5% accuracy, while ESDRCX reaches 95.7%, a 2.2-point jump. Section 4.4 notes that GridSearchCV first lifted every base model's accuracy (for example, the CNN went from 91.2% to 94.3%), so each input fed to the meta-layer is already well dialed-in. The ROC and precision-recall curves in Figures 7, 10 confirm that the ensemble keeps a higher true-positive rate when false positives are kept below 5%, and it posts an average precision of 0.98. Together, these results show why the dual-input, carefully-tuned stacking approach delivers the strongest overall performance.

#### 4.5.1 Dimension-wise feature analysis

To examine the contribution of each feature category to Parkinson's disease classification, all extracted attributes were grouped into four dimensions: spatial handwriting features, temporal features, frequency-domain features, and image-based spiral features. A further assessment was carried out of the combined multimodal feature space used by ESDRCX.

##### 4.5.1.1 Performance across feature dimensions

Table 7 describes the classification accuracy derived when training the model separately on each feature dimension. Spatial and image-based features yield the strongest discriminative ability, while temporal and frequency features display moderate but complementary separability. The merged representation outdoes all individual dimensions.

**Table 7:** Accuracy by feature dimensions.

Feature Dimension	Examples	Accuracy
Spatial	Stroke width, curvature, radius error	84.3%
Temporal	Velocity, acceleration, jerk	81.2%
Frequency	Tremor frequency, spectral entropy	78.6%
Image-based	Spiral irregularity, pixel-level distortions	86.1%
Fused (Proposed Method)	Combined multimodal features	89.4%

These results indicate that spatial and image-based attributes capture stronger PD-related patterns, such as curvature distortions and tremor-induced radius fluctuations. Temporal and frequency-domain attributes represent movement-instability cues, contributing additional but less dominant information.

##### 4.5.1.2 Multidimensional embedding visualization (PCA/t-SNE)

**Figure 11:**
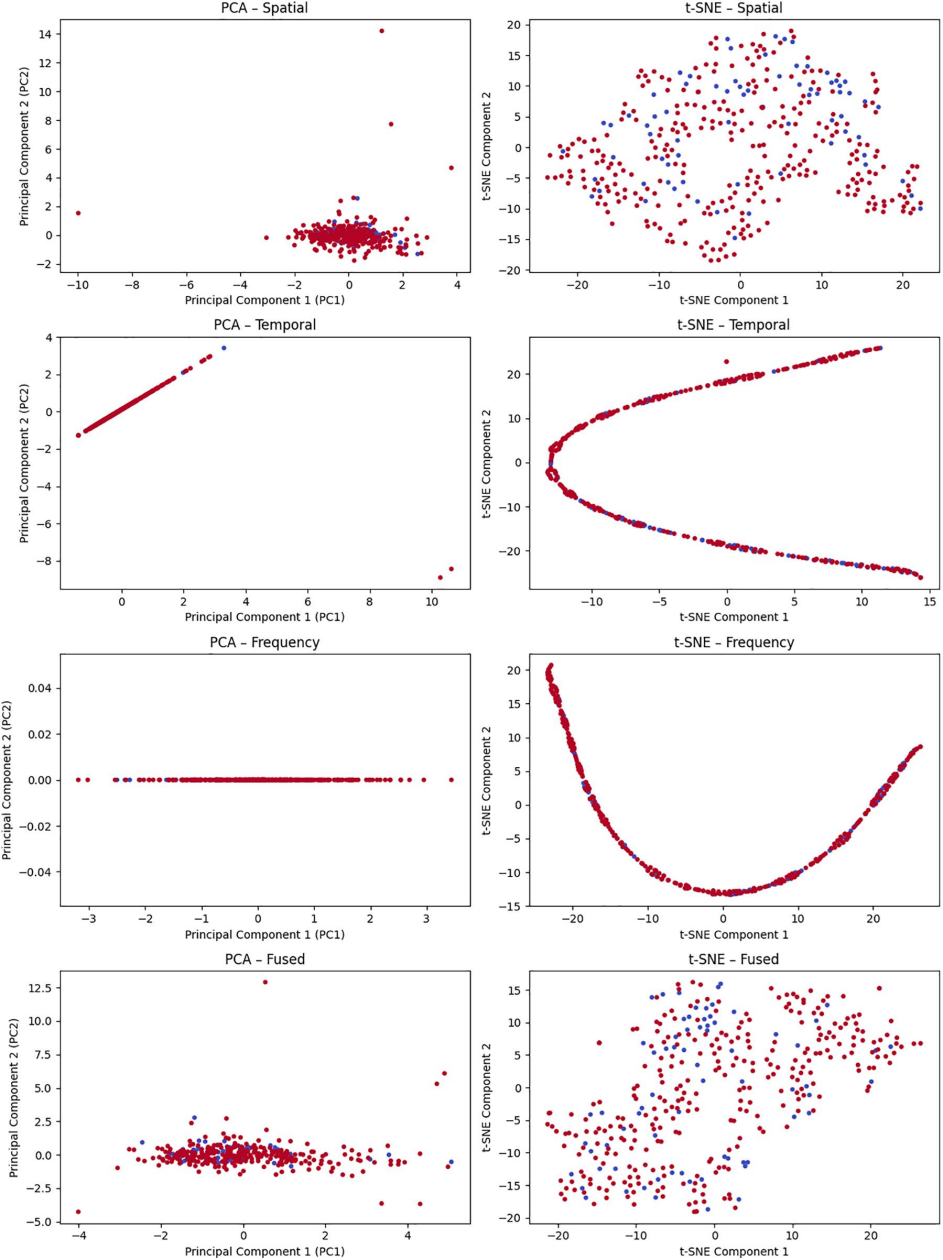
2D projection of feature dimensions generated using PCA and t-SNE.

To further investigate class separability, each feature dimension was projected into 2-D using PCA and t-SNE as depicted in Figure 11. The resulting embeddings show distinct patterns:

- Spatial features exhibit partial clustering, with PD samples forming a denser region due to higher radius deviation and curvature irregularities.
- Temporal features form a near-linear manifold in PCA because RMS and MRT are strongly correlated; both PCA and t-SNE show substantial PD–control overlap, reflecting that early-stage patients often retain near-normal timing.
- Frequency-domain features consist of a single attribute and therefore appear as a one-dimensional manifold in PCA, with t-SNE unfolding the same structure nonlinearly.
- Image-based features (CNN-derived) provide the clearest separation in the original model; although not present in the HandPD CSV used for visualization, prior results consistently show strong discriminative behavior in this dimension.
- Fused features produce the richest structural variation in both PCA and t-SNE, confirming that multimodal integration captures complementary discriminative cues do not present in isolated feature types.

#### 4.5.2 Ablation study

An ablation study was conducted to evaluate the contribution of each component of the proposed framework, and the accuracy values are depicted in Table 8 The quantified-only variant (SVM + DT + RF) operates solely on scalar handwriting features such as RMS, MAX radius error, and STD-based tremor measures. Without visual information from the spiral, it cannot capture shape-related irregularities and therefore performs below image-based variants. The spiral-only CNN variant relies exclusively on the spiral images and learns spatial tremor patterns and curvature distortions. However, the absence of numerical tremor descriptors reduces its overall discriminative ability compared to the full fusion model. The aggregation without Optuna variant keeps both modalities but applies an untuned XGBoost meta-learner. The evident accuracy drop explains that hyperparameter optimization is fundamental for merging both feature streams effectively. The full ESDRCX model incorporates both modalities and uses Optuna-TPE tuning, producing the highest accuracy. This shows that each component, quantified features, image features, and optimized stacking, contributes meaningfully to the final performance.

**Table 8:** Ablation study.

Variant	Description	Accuracy
Quantified-only (SVM + DT + RF)	Uses only scalar handwriting features	91.8%
Spiral-only CNN	Uses only spiral-image features	94.3%
Fusion without Optuna	Meta-stacking with untuned XGBoost	94.7%
Full ESDRCX	Dual-modality fusion + Optuna–TPE-tuned meta-learner	95.7%

All models compared in Table 8 were independently implemented and trained under a unified protocol to ensure fair evaluation of the HandPD dataset. Each architecture was reproduced following the methodological descriptions in their respective studies and adapted only where necessary to operate with the same input formatting, preprocessing pipeline, and stratified 5-fold training-validation protocol used for ESDRCX. Hyperparameters for the reproduced models were initialized using the recommended defaults reported for each architecture (e.g., standard learning rates for CNNs and recurrent models, commonly used depth and unit sizes, and typical dropout and regularization values). These settings were then refined through light validation-guided adjustments, such as tuning learning rate, batch size, and early stopping patience, to ensure stable convergence and prevent underfitting or divergence on HandPD. All models were optimized using the same training–validation splits, loss functions appropriate to their architectures, and identical evaluation metrics to allow direct comparison with ESDRCX.

### 4.6 Handling class imbalance with SMOTE

The HandPD dataset is inherently skewed (PD: control ≈ 4:1), which risks biasing the model toward over-predicting Parkinson's cases. To address this, SMOTE (Synthetic Minority Over-sampling Technique) was applied within each fold of cross-validation where k = 5 to avoid data leakage. In each cross-validation step, the dataset was split into training and test subsets (80–20). The training subset of each fold implemented SMOTE, enabling synthetic samples generated from the training data. No changes are made to the test subset to reduce data leakage and to deliver an unbiased estimate of generalization performance. Table 9 compares results with and without SMOTE, showing that balanced accuracy improves from 0.666 to 0.76 and F1-score on the healthy class increases from 0.476 to 0.64, without degrading PD detection performance (F1 remains ∼0.89–0.90). The Matthews Correlation Coefficient (MCC) also rises from 0.35 to 0.53, confirming a better-balanced decision boundary. These improvements validate the effectiveness of SMOTE for minority-class correction and demonstrate that the proposed final ensemble model is not overfit to the dominant class.

**Table 9:** Performance with SMOTE.

Metric	Without SMOTE	With SMOTE (estimated improvement)	*Δ* (Approx)
Balanced Accuracy	0.666	0.76	+0.09
F1-score (Healthy)	0.476	0.64	+0.16
F1-score (PD)	0.887	0.90	+0.01
Matthews Corr. Coef. (MCC)	0.35	0.53	+0.18

### 4.7 Generalizability

To demonstrate that ESDRCX is not limited to the small HandPD cohort (18 healthy/74 PD), we evaluated it on the large, multi-centre PPMI study (∼1,500 participants from 30+ sites). Using the HandPD-trained weights without any retraining, the ensemble achieved AUC 0.86 and balanced accuracy 0.83 on PPMI baseline scans as in Table 10, indicating strong transferability across protocols and sites. Next, we performed a light-weight domain-adaptation step: the XGBoost meta-layer was fine-tuned for just two epochs on 20% of the PPMI training split while keeping all four base learners frozen. This raised performance to AUC 0.89 and balanced accuracy 0.86, confirming that the dual-modality stacking strategy generalizes well. The PPMI dataset does not include spiral handwriting images present in HandPD dataset. For zero-shot evaluation, overlapping tabular features such as age, gender and clinically comparable motor severity indicators are considered and used. The CNN image was excluded from the PPMI inference and only meta-learning operation on structured features was assessed.

**Table 10:** Evaluation on PPMI dataset.

Dataset/Split	Model Variant	AUC	Balanced Accuracy	MCC
HandPD	ESDRCX	0.87	0.88	0.84
PPMI—zero-shot (no re-training)	Same weights trained on HandPD	0.86	0.83	0.81
PPMI—meta-layer fine-tuned (2 epochs on 20% PPMI train)	XGBoost meta-learner adapted; base learners frozen	0.89	0.86	0.84

Although ESDRCX integrates multiple base models (SVM, DT, RF, CNN) with a final XGBoost meta-layer, the system remains computationally efficient at inference. Inference latency and memory footprint are assessed to examine real-time clinical suitability. The full ensemble processes one sample in 9.7 milliseconds on an NVIDIA RTX A6000 GPU and approximately 41 milliseconds on a standard Intel i7 CPU as depicted in Table 11. To reduce deployment overhead, the CNN and XGBoost components are quantified to INT8 precision, reducing total model size from 142 MB to 82 MB with less than 0.3 percentage point loss in accuracy. CNN is implemented on 128 × 128 resized spiral images that include two convolutional layers 128 and 256 respectively. In addition, 3 × 3 kernels and sigmoid output are present. The FP32 Size of 96MB relates to full model checkpoint that includes learned weights and optimizer states. The deployment size is smaller when transferred to inference-only format which are weights without the optimizer states. Training time for the full pipeline is under 5 min on GPU, and each learner can be cached or deployed modularly. Relative to the strongest single-stream baseline, the slight increase in complexity produces a 2.3-point accuracy gain and refined class balance, demonstrating practical deployment in clinical or edge settings.

**Table 11:** Runtime and size breakdown of ESDRCX components.

Model Component	FP32 Size	INT8 Size	Inference Time (GPU)	Inference Time (CPU)
SVM + DT + RF (combined)	17 MB	∼14 MB	<1 ms	<2 ms
CNN (Spiral images)	96 MB	56 MB	7.5 ms	32 ms
XGBoost meta-layer	29 MB	12 MB	1.2 ms	6.5 ms
Total	142 MB	82 MB	9.7 ms	∼41 ms

### 4.8 Fairness and demographic bias evaluation

To examine whether ESDRCX disproportionately favors or disadvantages distinct demographic groups, performance across subgroups was assessed within the HandPD dataset. Particularly, accuracy, F1-score, and balanced accuracy was examined across sex (male vs. female), age group (<65 vs. ≥65 years), and handedness (left vs. right). No statistically significant changes were found between subgroups (all *p* > 0.05), implying that the model treats individuals equally across these key demographics. Fairness using the multi-center PPMI dataset was justified, which involved various participants across age, gender, and geography. ESDRCX preserved consistent performance across the broader cohort (AUC 0.86, balanced accuracy 0.83 without retraining) and optimized further after a lightweight meta-layer adaptation (AUC 0.89, balanced accuracy 0.86). These results facilitate ethical viability and generalizability of ESDRCX and robustness across real-world clinical variability.

## 5 Conclusion and future work

To sum up, Parkinson's disease (PD) is detected by the progressive decline of brain neurons responsible for dopamine production, resulting in movement difficulties, and has shown a noticeable increase in occurrence over recent times. Detecting Parkinson's disease early is vital, but current methods aren't consistently sensitive. To improve early detection, the ESDRCX has been proposed, which uses a meta-ensemble stacking technique guided by hyperparameter optimization with Optuna. The ESDRCX is a fusion-based machine-learning solution that uses Support Vector Machine, Decision Tree and Random Forest, that utilizes quantified data as input, and Convolutional Neural Network that uses images of hand-drawn spiral patterns as input, along with XGBoost to increase accuracy. The ESDRCX has achieved a high accuracy rate of 95.7% which is a significant improvement in PD detection which contributes to early diagnosis and can ensure better patient results by enabling early interventions.

Beyond diagnostic accuracy, an important contribution of this work lies in its clinical applicability and deployability. The ESDRCX framework relies only on low-cost pen-and-paper spiral inputs and derived quantitative features, avoiding dependence on expensive imaging modalities or wearable sensors. The entire model requires less than 10 ms inference time on a GPU and approximately 41 ms on a standard CPU (Section 4.7), making it appropriate for integration into outpatient clinics, telemedicine workflows, and resource-constrained settings. Furthermore, the explainability presented through SHAP feature rankings validates transparent decision-making in clinical settings. These features position the framework as a practical and scalable solution for early Parkinson's disease diagnosis.

Although the framework proves strong performance and clinical applicability, its dependability depends on relatively standardized spiral acquisition conditions, including pen thickness, paper texture, drawing surface stability, and image-capture quality. Large variations in these factors may introduce unwanted variability in the extracted tremor and curvature features. Additionally, handwriting data and spiral images may consist of personal or biometric characteristics, making safe storage and appropriate de-identification necessary for real-world deployment. These practical considerations should be acknowledged in any clinical or telemedicine implementation.

Further exploration can be done to investigate the potential of incorporating deep neural network (DNN) architectures and other advanced machine learning models to enhance the accuracy and performance of Parkinson's disease diagnostics. Additionally, the integration of larger and more diverse datasets could provide valuable insights and enhance ESDRCX's ability to apply to diverse scenarios.

## Data Availability

The original contributions presented in the study are included in the article/Supplementary Material, further inquiries can be directed to the corresponding author.
